# Intranasal delivery of a small-molecule ErbB inhibitor promotes recovery from acute and late-stage CNS inflammation

**DOI:** 10.1172/jci.insight.154824

**Published:** 2022-04-08

**Authors:** Mathias Linnerbauer, Lena Lößlein, Oliver Vandrey, Thanos Tsaktanis, Alexander Beer, Ulrike J. Naumann, Franziska Panier, Tobias Beyer, Lucy Nirschl, Joji B. Kuramatsu, Jürgen Winkler, Francisco J. Quintana, Veit Rothhammer

**Affiliations:** 1 Department of Neurology, University Hospital Erlangen, Friedrich–Alexander University Erlangen–Nürnberg, Erlangen, Germany.; 2 Department of Neurology, Rechts der Isar Hospital, Technical University of Munich, Munich, Germany.; 3 Department of Molecular Neurology, Friedrich–Alexander University Erlangen–Nürnberg, Erlangen, Germany.; 4 Ann Romney Center for Neurologic Diseases, Brigham and Women’s Hospital, Harvard Medical School, Boston, Massachusetts, USA.; 5 Broad Institute of MIT and Harvard, Cambridge, Massachusetts, USA.

**Keywords:** Autoimmunity, Neuroscience, Autoimmune diseases, Multiple sclerosis, Neurodegeneration

## Abstract

Multiple sclerosis (MS) is an autoimmune inflammatory disease of the CNS that is characterized by demyelination and axonal degeneration. Although several established treatments reduce relapse burden, effective treatments to halt chronic progression are scarce. Single-cell transcriptomic studies in MS and its animal models have described astrocytes and their spatial and functional heterogeneity as important cellular determinants of chronic disease. We combined CNS single-cell transcriptome data and small-molecule screens in primary mouse and human astrocytes to identify glial interactions, which could be targeted by repurposing FDA-approved small-molecule modulators for the treatment of acute and late-stage CNS inflammation. Using hierarchical in vitro and in vivo validation studies, we demonstrate that among selected pathways, blockade of ErbB by the tyrosine kinase inhibitor afatinib efficiently mitigates proinflammatory astrocyte polarization and promotes tissue-regenerative functions. We found that i.n. delivery of afatinib during acute and late-stage CNS inflammation ameliorates disease severity by reducing monocyte infiltration and axonal degeneration while increasing oligodendrocyte proliferation. We used unbiased screening approaches of astrocyte interactions to identify ErbB signaling and its modulation by afatinib as a potential therapeutic strategy for acute and chronic stages of autoimmune CNS inflammation.

## Graphical abstract

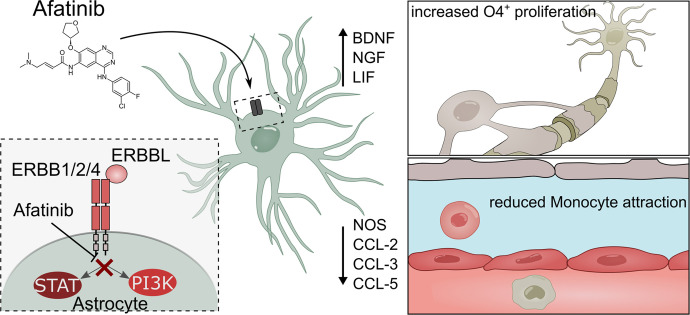

## Introduction

Multiple sclerosis (MS) is a chronic autoimmune inflammatory disease of the CNS (1, 2). It is characterized by the infiltration of autoreactive immune cells into the CNS, followed by demyelination and neurodegeneration (1). MS predominantly presents during early adulthood but progresses throughout the patient’s lifetime, causing increasing individual and socioeconomic burdens (2, 3). Based on clinical criteria, 3 major subtypes of MS can be differentiated. Approximately 80% to 90% of all patients are initially diagnosed with relapsing-remitting MS (RRMS), which is characterized by recurring neurological deficits defined by lesion localization followed by a remission period of clinical recovery (1). The majority of patients with RRMS eventually develop secondary progressive MS, a disease phase characterized by progressive and irreversible neurological decline (1). Finally, approximately 10% of patients with MS are initially diagnosed as having a primary progressive subtype of MS, which is characterized by continuous neurological deterioration in the absence of defined relapses (1, 4).

Important progress has been made in recent years in the treatment of RRMS, but treatment strategies for the progressive phase of the disease remain sparse. Indeed, RRMS pathology can be effectively controlled by agents targeting peripheral immune responses, but MS progression is mostly unresponsive to these immunotherapeutic approaches. In this context, astrocytes and microglia have gained increasing attention as key modulators of chronic CNS inflammation (5, 6). However, little is known about the mechanisms that regulate beneficial and disease-promoting astrocyte and microglial activities (7).

Single-cell transcriptomic profiling in combination with high-throughput flow cytometry screens has identified novel astrocyte subsets, which, depending on time and context, drive or suppress CNS inflammation (8, 9). These opposing functions of reactive astrocytes are shaped by their microenvironment and interactions with CNS-resident and CNS-infiltrating cells (9, 10). In this study, we used public, single-cell RNA-Seq (scRNA-Seq) data to infer receptor–ligand interactions of human and mouse astrocytes regulated in acute and late-stage CNS inflammation, which ultimately allowed us to identify pathways targetable by FDA-approved small-molecule modulators and recombinant proteins. Hierarchical in vitro screening of multiple small-molecule inhibitors in combination with functional studies demonstrated that blockage of ErbB signaling by the small-molecule tyrosine kinase inhibitor (TKI) afatinib reduced pathogenic astrocyte polarization and promoted CNS-protective functions in mouse and human primary astrocytes. Finally, i.n. drug delivery of afatinib during acute and late-stage CNS inflammation ameliorated disease severity and promoted recovery in a preclinical model of MS. Together, these approaches may guide the development of therapeutic strategies for MS, particularly its progressive phase, for which efficient therapies are limited.

## Results

### Screening of astrocyte interactions identifies druggable targets in CNS inflammation.

**Figure 1:**
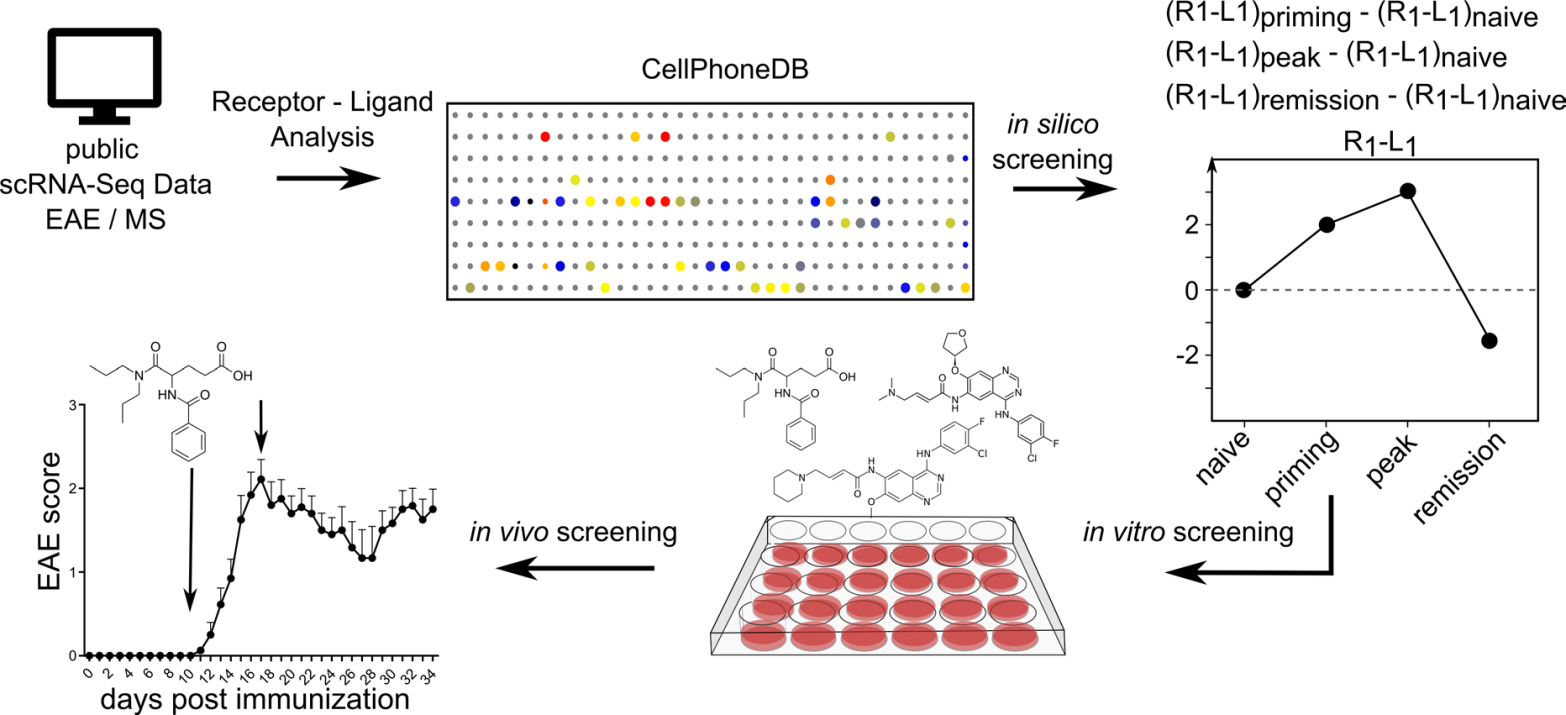
Schematic workflow. In silico identification of inferred cell-cell interactions using CellPhoneDB, followed by in vitro screening and in vivo i.n. delivery in a mouse model of MS. R1, receptor 1; L1, ligand 1.

Astrocytes have gained increasing attention in acute and, particularly, chronic stages of autoimmune CNS inflammation, for which efficient therapeutic strategies are limited. To identify disease-modifying agents acting on astrocytes, particularly during chronic CNS inflammation, we sought to identify cell-cell interactions between astrocytes and other cell types specifically regulated over the course of experimental autoimmune encephalomyelitis (EAE), the animal model of MS. For this, we used a scRNA-Seq data set recently published by Wheeler et al. (8) in which the authors analyzed 24,275 CNS cells isolated during the naive, priming, peak, and late chronic phases of EAE. We used the CellPhoneDB repository to infer receptor–ligand cell–cell interactions between astrocytes and other cell types over the course of EAE, and we investigated selected receptor–ligand interactions of interest using hierarchical in vitro and in vivo approaches (Figure 1) (8, 11). As expected, the priming and peak phases of EAE were characterized by an increase in interactions involving peripheral immune cells (mostly myeloid cells and T cells) and CNS resident cells such as glial cells, whereas cell-cell interactions in the chronic phase were more diverse (Supplemental Figure 1; supplemental material available online with this article; https://doi.org/10.1172/jci.insight.154824DS1).

**Figure 2:**
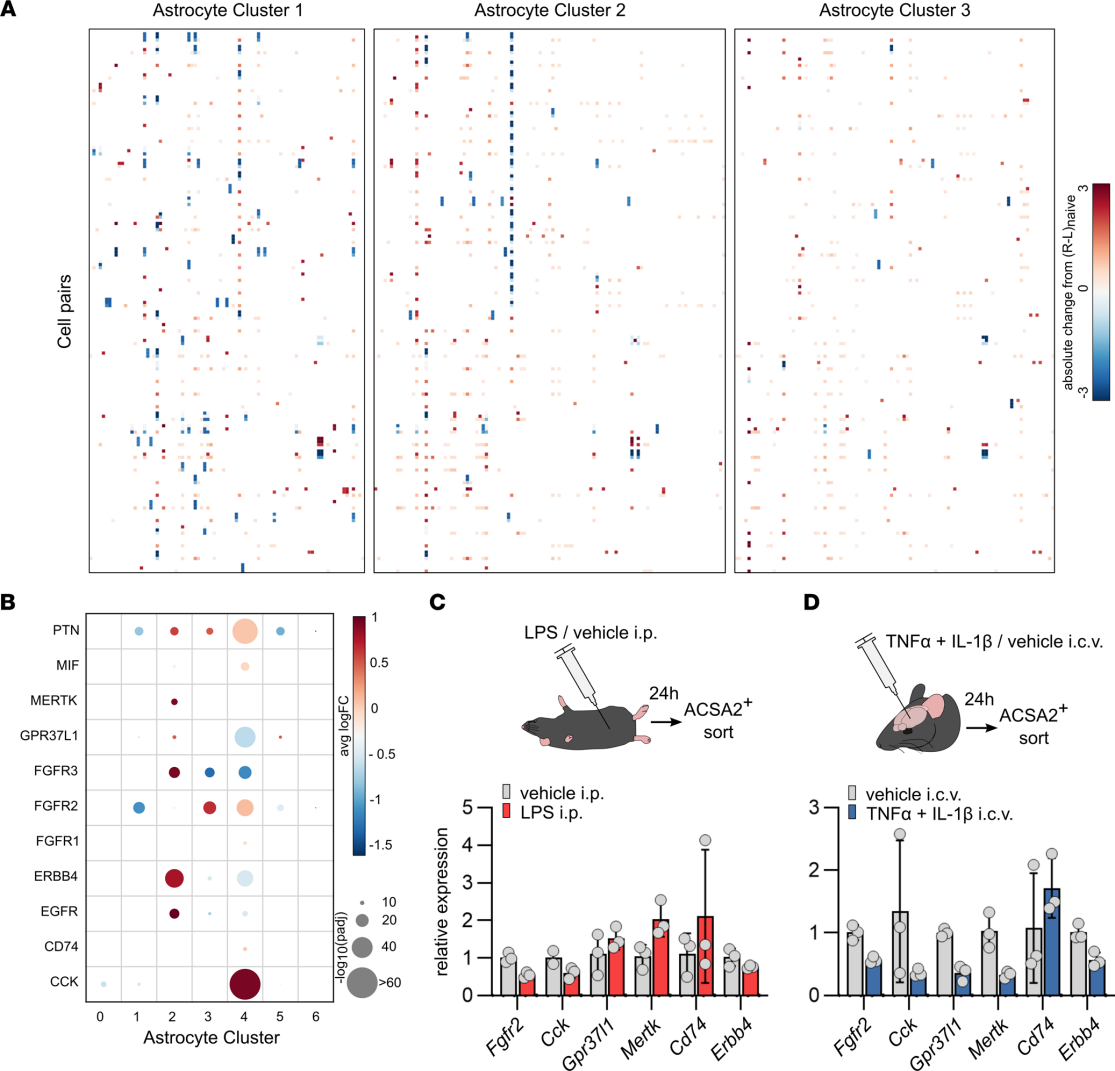
Bioinformatic identification of astrocyte-cell interactions. (**A**) Receptor–ligand pairs between astrocyte-cell pairs regulated throughout the course of EAE (naive, onset, peak, remission). Single-cell data were obtained from Wheeler et al. (8). (**B**) Selected receptor–ligand pairs and their expression in cortical astrocyte subclusters of patients with MS. Data were obtained from Wheeler et al. (8). (**C**) Expression of selected receptors in ACSA2^+^ cortical astrocytes after peripherally induced neuroinflammation, quantified by reverse transcriptase qPCR (RT-qPCR). LPS injected mice, *n* = 3; mice injected with vehicle (PBS), *n* = 3. (**D**) Expression of selected receptors in ACSA2^+^ cortical astrocytes after cytokine-induced neuroinflammation, quantified by RT-qPCR. Mice injected with cytokines (TNF-α and IL-1β), *n* = 3; mice injected with vehicle (PBS), *n* = 3. Data reported as mean ± SD. Avg, average; FC, fold change.

Because we aimed to identify druggable signaling pathways regulating astrocyte functions during chronic inflammation, we focused on receptor–ligand pairs between astrocytes and CNS resident and infiltrating cell types and computed the absolute change in receptor–ligand co-expression compared with the naive state (Figure 1 and Figure 2A). Using this approach, we identified 183 astrocyte receptor–ligand pairs regulated over the course of the disease; a positive change indicated that the receptor–ligand interaction was more abundant during the respective stage than in the naive state (Figure 2A). Notably, astrocyte clusters predefined in the study by Wheeler et al. (8) differed in number and type of inferred cell-cell interactions throughout the disease (Supplemental Figure 2, A and B). Of the 183 regulated receptor–ligand pairs, 37 were shared among all astrocytes, indicating signaling pathways conserved in astrocyte function independent of their transcriptional and functional subtype (Supplemental Figure 2A).

Next, we curated and cross-referenced receptor–ligand pairs up- or downregulated during remission, which could be targeted by disease-modifying compounds approved or under approval in the context of other human diseases (Supplemental Table 1). Interestingly, the selected receptors and ligands were differentially regulated within previously defined astrocyte subpopulations (8) (Supplemental Figure 2, C and D), suggesting that their therapeutic modulation may affect individual astrocyte activation states differentially.

To confirm the relevance of these receptor–ligand interactions in MS, we analyzed public scRNA-Seq data sets (12) of human cortical astrocyte clusters in patients with MS and in control participants. Indeed, we detected differential regulation of the selected receptors and ligands in distinct astrocyte subsets in MS, suggesting that these pathways may be relevant in the modulation of astrocyte pathogenicity in MS (Figure 2B).

Finally, we aimed to validate our observations and confirm the regulation of the selected receptors in activated astrocytes in disease-specific contexts, such as peripherally induced neuroinflammation as opposed to primarily CNS-intrinsic inflammation (Figure 2, C and D). Although LPS-induced neuroinflammation (Figure 2C) depends on peripheral activation of the immune system, blood–brain barrier breakdown, and penetration of peripheral mediators and immune cells into the CNS, i.c.v. injection of TNF-α and IL-1β, 2 proinflammatory cytokines involved in the pathogenesis of MS and known activators of astrocytes (13), resulted in acute CNS-intrinsic inflammation (Figure 2D). Indeed, both paradigms induced significant regulation of the selected astrocyte receptors (Figure 2, C and D). Yet, *Gpr37l1* and *Mertk* were inversely regulated, pointing toward a complex but potentially meaningful regulation of these pathways under peripherally or centrally induced CNS inflammation.

### Bioinformatically selected agents regulate inflammatory astrocyte signaling.

**Figure 3:**
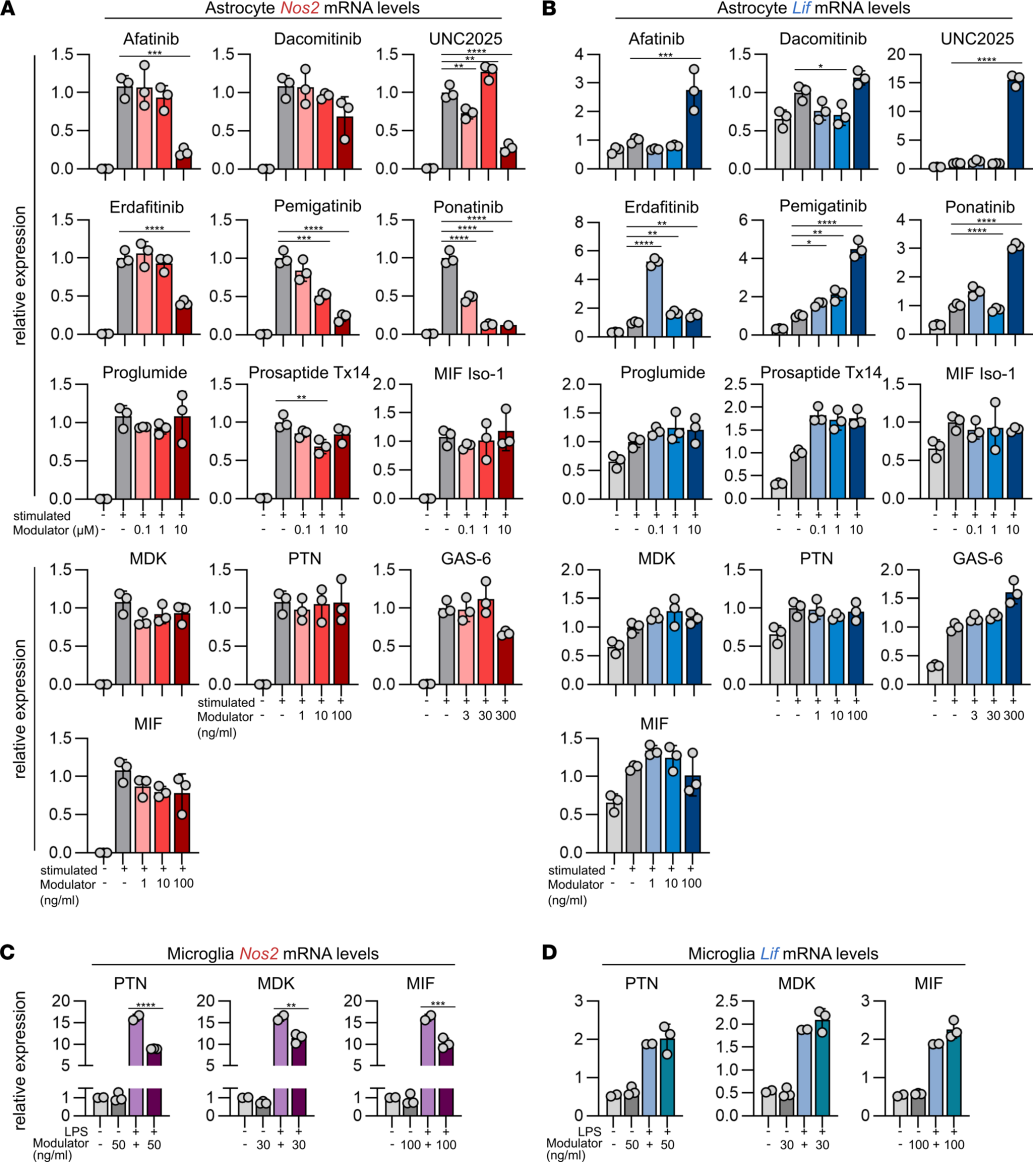
In vitro screening of selected modulators in primary mouse astrocytes and microglia. (**A**) *Nos2* and (**B**) *Lif* expression, quantified by reverse transcriptase qPCR (RT-qPCR) in primary mouse astrocytes after stimulation with TNF-α and IL-1β in combination with increasing concentrations of the selected modulators; data are representative for *n* = 2 independent experiments with *n* = 3 replicates each per group. The respective concentrations are provided in Methods. One-way ANOVA with Dunnett’s multiple comparisons test; data are reported as mean ± SD. (**C** and **D**) *Nos2* (**C**) and *Lif* (**D**) expression, quantified by RT-qPCR in primary mouse microglia after stimulation, or not, with LPS in combination with increasing concentrations of cytokines produced by astrocytes. Data are representative for *n* = 2 independent experiments with *n* = 3 replicates each per group. The respective concentrations are provided in Methods. One-way ANOVA with Dunnett’s multiple comparisons test; data are reported as mean ± SD. *P < 0.05; **P < 0.01; ***P < 0.001; ****P < 0.0001.

To validate the impact of the receptor modulators and astrocyte-derived ligands discovered in the bioinformatic analyses of glial pathogenic or protective activities, we exposed primary mouse astrocytes and microglia to proinflammatory stimuli in the presence of the selected agents. Tissue-protective glial polarization, as exemplified by downregulation of proinflammatory *Nos2* and upregulation of protective *Lif,* was induced by EGFR modulators afatinib and dacomitinib; FGF receptor (FGFR) modulators erdafitinib, ponatinib, and pemigatinib; as well as the GPR37L1 and GPR37 agonist prosaptide TX14, the MER/FLT TKI UNC2025, and growth-arrest-specific gene-6 (GAS6) (Figure 3, A and B); whereas none of the other candidates showed an effect on the expression of *Nos2* or *Lif* in astrocytes.

In addition to the small-molecule inhibitors targeting receptor signaling in activated astrocytes, we exposed primary microglia to the astrocyte-derived growth factors PTN, MIF, and MDK, which were part of the differentially regulated astrocyte-derived ligand–receptor interactions over the course of EAE (Supplemental Figure 2A). Indeed, all 3 growth factors induced downregulation of proinflammatory *Nos2* expression in microglia, but they had no effect on *Lif* expression, collectively suggesting that they may be part of a protective astrocyte signature (Figure 3, C and D).

### Afatinib, UNC2025, and pemigatinib modulate astrocyte pathogenic functions.

**Figure 4:**
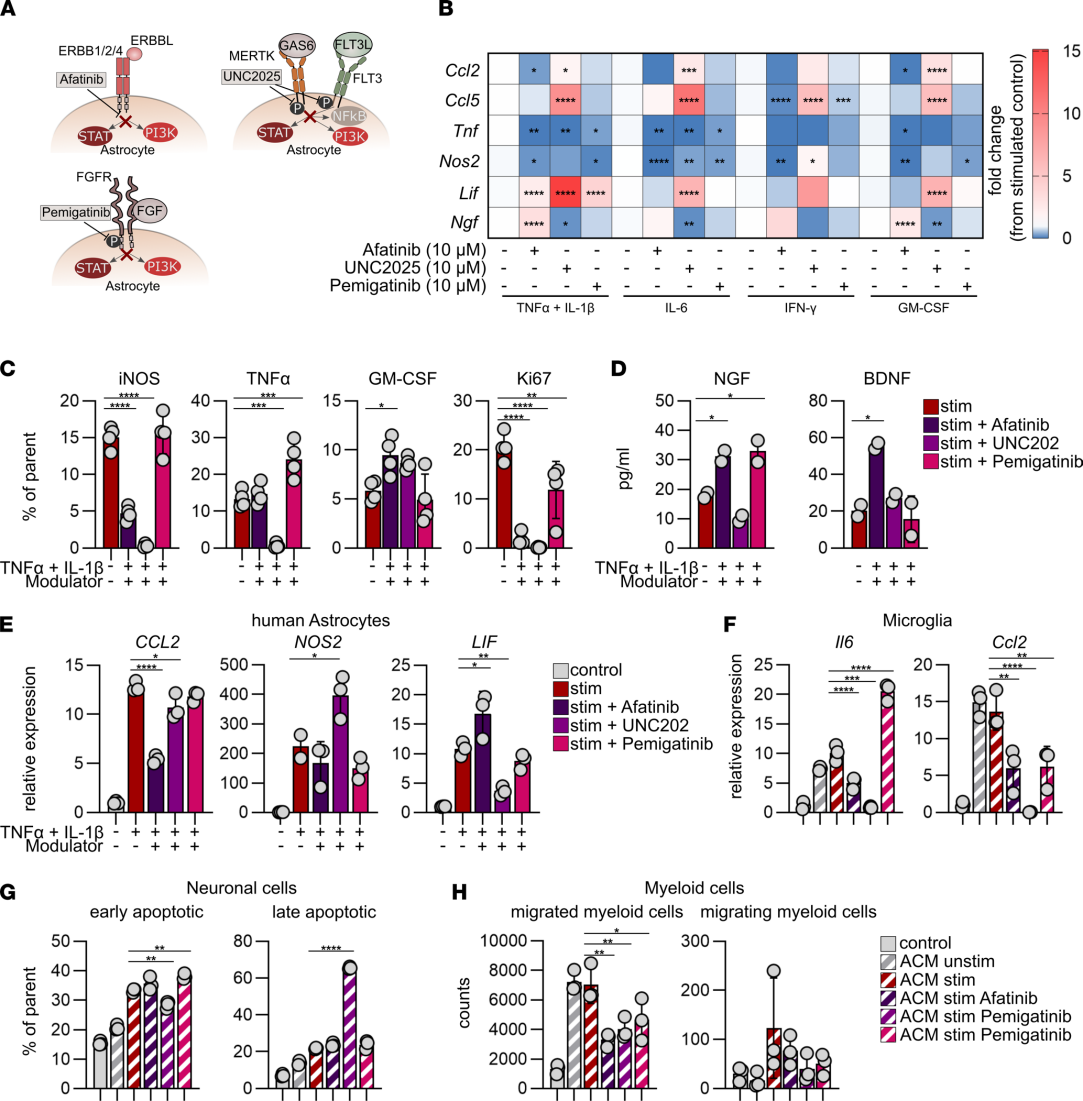
In vitro validation of selected modulators in primary mouse astrocytes. (**A**) Schematic mechanism of action of afatinib, UNC2025, and pemigatinib. (**B**) *Ccl2*, *Ccl5*, *Tnf*, *Nos2*, *Lif*, and *Ngf* expression, quantified by reverse transcriptase qPCR (RT-qPCR) in primary mouse astrocytes after stimulation (stim) with TNF-α and IL-1β, IL-6, IFN-γ, or GM-CSF with or without afatinib, UNC2025, and pemigatinib; *n* = 4 per group. Two-way ANOVA with Dunnett’s multiple comparisons test. (**C**) iNOS, TNF-α, GM-CSF, and Ki-67 were quantified by intracellular flow cytometry of primary mouse astrocytes after stim with TNF-α and IL-1β with or without afatinib, UNC2025, and pemigatinib. Data are representative for *n* = 2 independent experiments with *n* = 4 per group. One-way ANOVA with Dunnett’s multiple comparisons test; data are reported as mean ± SD. (**D**) ELISA measurement of NGF and BDNF produced by primary mouse astrocytes after stim with TNF-α and IL-1β with or without afatinib, UNC2025, and pemigatinib; *n* = 2. One-way ANOVA with Dunnett’s multiple comparisons test; data are reported as mean ± SD. (**E**) *CCL2*, *NOS2*, and *LIF* expression quantified by RT-qPCR in human astrocytes with or without stim with TNF-α and IL-1β with or without afatinib, UNC2025, and pemigatinib; *n* = 4. One-way ANOVA with Dunnett’s multiple comparisons test; data are reported as mean ± SD. (**F**) Relative expression of *Il6*, and *Ccl2* in primary mouse microglia quantified by RT-qPCR after stim with ACM derived from unstimulated and stim (TNF-α and IL-1β) primary mouse astrocytes with or without afatinib, UNC2025, and pemigatinib; *n* = 3. One-way ANOVA with Dunnett’s multiple comparisons test; data are reported as mean ± SD. (**G**) Annexin V propidium iodide apoptosis assay of neuronal N2A cells stimulated with ACM; *n* = 3. Cells were categorized as in early or late apoptosis. One-way ANOVA with Dunnett’s multiple comparisons test; data are reported as mean ± SD. (**H**) Migration assay of CD11b^+^ myeloid cells stimulated with ACM; *n* = 3. One-way ANOVA with Dunnett’s multiple comparisons test; data are reported as mean ± SD. *P < 0.05; **P < 0.01; ***P < 0.001; ****P < 0.0001.

Next, we focused on the most potent candidates in each pathway system based on the results of our in vitro transcriptional screen, selecting afatinib as an EGFR inhibitor, pemigatinib as an FGFR modulator, and UNC2025 as a suppressor of MER/FLT signaling to determine their impact on protective astrocyte polarization at the transcriptional, protein, and functional levels (Figure 4A). Afatinib successfully induced antiinflammatory polarization in preactivated astrocytes, characterized by the downregulation of proinflammatory genes (*Csf2*, *Tnf*, *Ccl2, Ccl3*, *Ccl5*, *Cxcl10*) and upregulation of the antiinflammatory genes *Ngf* and *Bdnf* in response to stimulation with TNF-α plus IL-1β, IL-6, IFN-γ, and GM-CSF (Figure 4B and Supplemental Figure 3, A–D). UNC2025 treatment resulted in increased expression of proinflammatory markers (*Ccl2*, *Ccl5*, *and Csf2*), whereas treatment with pemigatinib attenuated proinflammatory signaling (*Csf2*, *Tnf*, *Ccl2*, *Ccl5*, *Cxcl10*) (Figure 4B and Supplemental Figure 3, A–D).

These observations were further confirmed at the protein level by intracellular flow cytometry and ELISA measurements (Figure 4, C and D, and Supplemental Figure 4A). Both afatinib and UNC2025 significantly reduced astrocyte proliferation, as previously described (14–16). To exclude that the reduction in proliferation was associated with increased apoptosis of astrocytes, we quantified their survival by annexin V–propidium iodide and amine-dependent live-dead staining. Afatinib and pemigatinib showed no effect on cellular survival; however, UNC2025 treatment resulted in increased apoptosis (Supplemental Figure 4, B and C).

To validate the relevance of our findings for MS, we exposed primary human astrocytes to proinflammatory conditions in the presence of afatinib, UNC2025, or pemigatinib and subsequently analyzed their expression of proinflammatory and tissue-regenerative markers. Indeed, afatinib treatment decreased *CCL2* and increased *LIF* expression, matching our findings in murine in vitro settings, whereas UNC2025 exhibited limited protective effects only (Figure 4E and Supplemental Figure 4D).

Based on the anti-inflammatory effects of afatinib, UNC2025, and pemigatinib on astrocytes, we next investigated how these alterations affect other CNS-resident and peripheral cell types on a functional level. For this, we collected conditioned medium from primary astrocytes (ACM) pre-exposed to proinflammatory stimuli in the presence of afatinib, UNC2025, or pemigatinib, and we examined how treatment-dependent alterations in astrocyte pathogenicity modulate the immunological functions of microglia, survival of neuronal cells, and the migratory behavior of myeloid cells (Figure 4, E–H).

ACM from both afatinib- and UNC2025-treated astrocytes suppressed proinflammatory gene expression in primary microglia; ACM derived from pemigatinib-stimulated astrocytes failed to produce a consistent effect (Figure 4F). Similar effects were observed after direct treatment of microglia with afatinib, UNC2025, and pemigatinib, demonstrating distinct effects of pemigatinib on astrocytes and microglia (Supplemental Figure 4E). Neuronal survival was largely unaffected by ACM derived from afatinib- and pemigatinib-treated astrocytes, whereas ACM derived from UNC2025-treated astrocytes resulted in apoptosis of neuronal cells (Figure 4G and Supplemental Figure 4F).

Because astrocytes participate in the recruitment of proinflammatory myeloid cells during acute CNS inflammation, we investigated the effect of ACM derived from afatinib-, UNC2025-, and pemigatinib-treated astrocytes on the migratory behavior of CD11b^+^ myeloid cells. Indeed, treatment with afatinib, UNC2025, and pemigatinib successfully diminished the capacity of reactive astrocytes to recruit myeloid cells, matching our previous observations of reduced chemokine expression (Figure 4H).

Collectively, these data demonstrate that ErbB blockage by afatinib reduces pathogenic mechanisms in reactive astrocytes, which, in turn, attenuate proinflammatory signaling in microglia and reduce myeloid cell migration. This was also seen to a lesser extent after FGFR blockage by pemigatinib. Despite a positive net effect on microglia and the migratory behavior of myeloid cells, UNC2025 treatment of astrocytes induced proinflammatory gene expression and promoted neuronal death, which led us to exclude it from the rest of our investigation.

Overall, these results suggest that particularly ErbB blockade by afatinib and potentially FGFR modulation by pemigatinib may be promising for suppression of proinflammatory astrocyte responses while, at the same time, promoting protective mechanisms in murine and human systems.

### Delivery of afatinib i.n. ameliorates acute autoimmune CNS inflammation.

**Figure 5:**
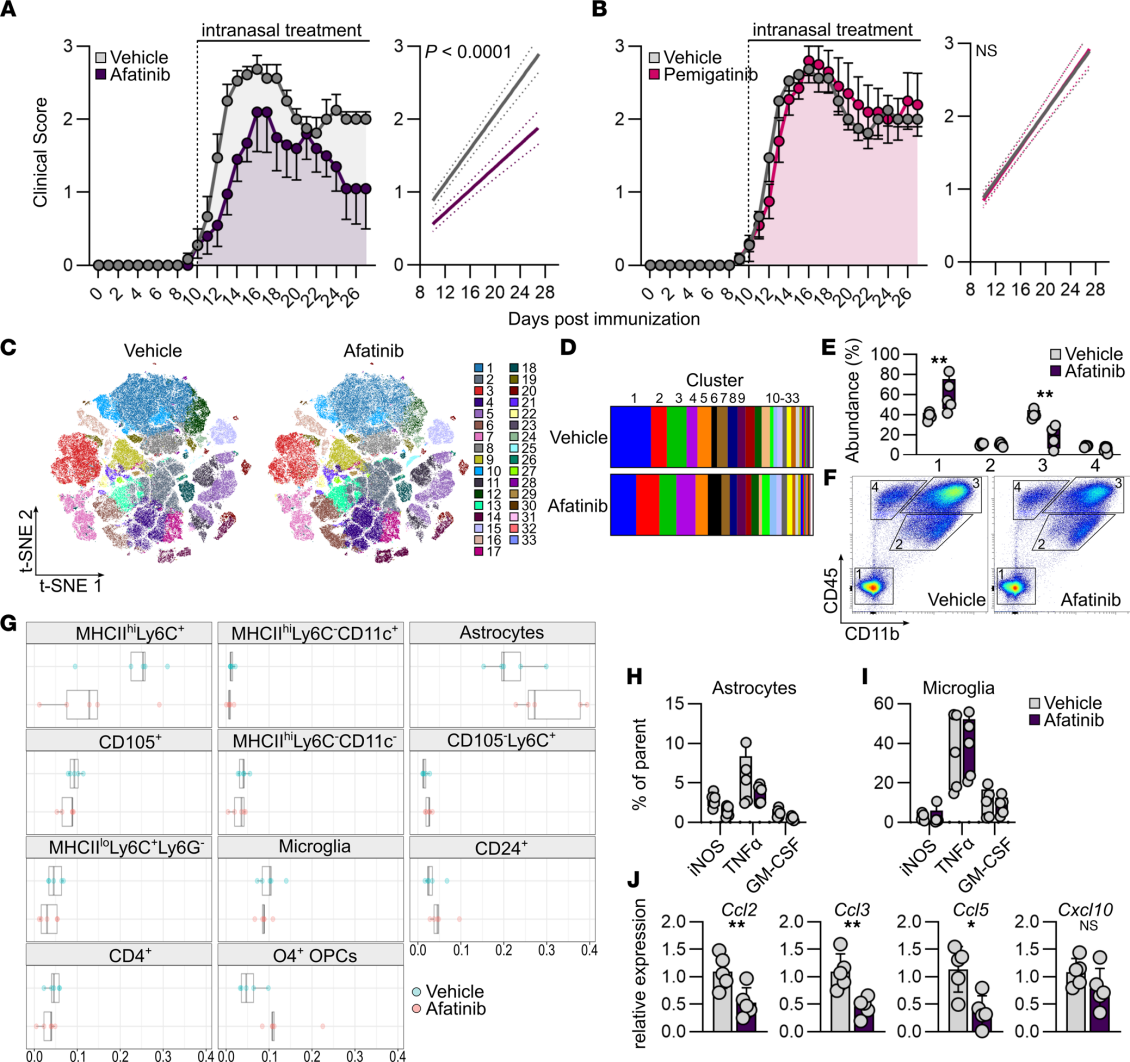
Therapeutic potential of i.n. afatinib and pemigatinib application in acute EAE. (**A** and **B**) Clinical course (left) and linear regression analysis (right) of EAE in mice after daily i.n. treatment with vehicle, afatinib (**A**), or pemigatinib (**B**) starting from symptom onset. The experiment was repeated twice. Vehicle (PBS), *n* = 10; afatinib, *n* = 10; pemigatinib, *n* = 10. Data are reported as mean ± SEM. (**C**) Unsupervised clustering t-distributed stochastic neighbor embedding (t-SNE) plot overlaid with cluster and their abundance (**D**) identified by unsupervised PhenoGraph clustering of high-dimensional flow cytometry data obtained from CNS (brain and spinal cord) tissue at peak of disease from mice treated with afatinib (*n* = 5) or vehicle (*n* = 5). (**E** and **F**) Quantification (**E**) and scatter plots (**F**) of CD45^–^CD11b^–^, CD45^int^CD11b^+^, CD45^hi^CD11b^+^, and CD45^+^CD11b^–^ cell populations in the CNS of mice treated with afatinib (*n* = 5) or vehicle (*n* = 5) at peak of disease. Two-way ANOVA with Šidák’s multiple comparisons test. (**G**) Significant differences in CNS-resident and -infiltrating cell populations in mice treated with afatinib (*n* = 5) or vehicle (*n* = 5) at peak of disease, identified by SAM analysis (29) (FDR cutoff, 0.1). (**H** and **I**) Intracellular flow cytometry quantification of iNOS, TNF-α, and GM-CSF in astrocytes (**H**) and microglia (**I**) in mice treated with afatinib (*n* = 5) or vehicle (*n* = 5) at peak of disease. (**J**) Relative expression of *Ccl2*, *Ccl3*, *CCl5*, and *Cxcl10* in ACSA2^+^-sorted astrocytes after i.n. treatment with afatinib (*n* = 5) or vehicle (*n* = 5). Unpaired *t* test with Welch’s correction. Data are reported as mean ± SD. *P < 0.05; **P < 0.01.

To investigate the therapeutic potential of afatinib and pemigatinib in vivo, we induced EAE in WT B6 mice by immunization with the myelin oligodendrocyte glycoprotein (MOG) epitope MOG_35–55_ in CFA, followed by injection of pertussis toxin. Starting after onset of symptoms, once autoreactive immune cells began to transgress into the CNS, we administered afatinib, pemigatinib, or vehicle i.n. daily. Administration of afatinib i.n. significantly ameliorated disease severity during the acute and late-stage phase, whereas no effect of pemigatinib on the disease course was evident (Figure 5, A and B).

Dimensionality reduction followed by unsupervised clustering of CNS cells analyzed by high-dimensional flow cytometry revealed significant changes in the cellular composition following treatment with afatinib compared with the control at peak of disease, which were mostly attributed to CD45^hi^CD11b^+^ myeloid cells and CD45^–^CD11b^–^ cells (Figure 5, C–F, and Supplemental Figure 5, A and B). Using significance analysis of microarray, a statistical method used for finding significant features from input data described by a response variable, we identified a significant decrease in MHC^hi^Ly6C^+^ monocytes, concomitant with an increase in O4^+^ oligodendrocytes and astrocytes, following treatment with afatinib (Figure 5G and Supplemental Figure 5, C–F). This finding was in line with an attenuated proinflammatory phenotype of astrocytes, but not microglia, together with a reduction in their chemoattractant capacities (Figure 5, H–J and Supplemental Figure 6A). In addition, we observed no difference in the inflammatory phenotype of CNS-infiltrating and spleen-resident myeloid cells or lymphocytes, indicating that afatinib does not directly alter their peripheral induction or inflammatory functions (Supplemental Figure 6, B–G).

In accordance with our in vitro observations, these data demonstrate that afatinib effectively regulates proinflammatory astrocyte signaling by reducing the expression of monocyte-attracting chemokines and other proinflammatory factors. These effects, at least to some extent, may ameliorate acute autoimmune CNS inflammation.

### Afatinib reduces late-stage CNS inflammation.

Because astrocytes are key players in chronic stages of CNS inflammation, for which current therapeutic strategies are limited, we evaluated the therapeutic potential of ErbB blockade during late-stage neuroinflammation as compared with treatment before symptom onset. Indeed, we observed upregulation of EGFR on astrocytes during late stages of CNS inflammation, indicating they may be more susceptible to afatinib treatment during progressive disease (Supplemental Figure 7, A and B). To evaluate early- as well as late-stage effects of afatinib for the resolution of CNS inflammation, we induced EAE in WT B6 mice, as described previously in Results, and started i.n. delivery of afatinib at symptom onset or after peak of disease, representing interventions clinically relevant for the treatment of acute and late stages of MS.

**Figure 6:**
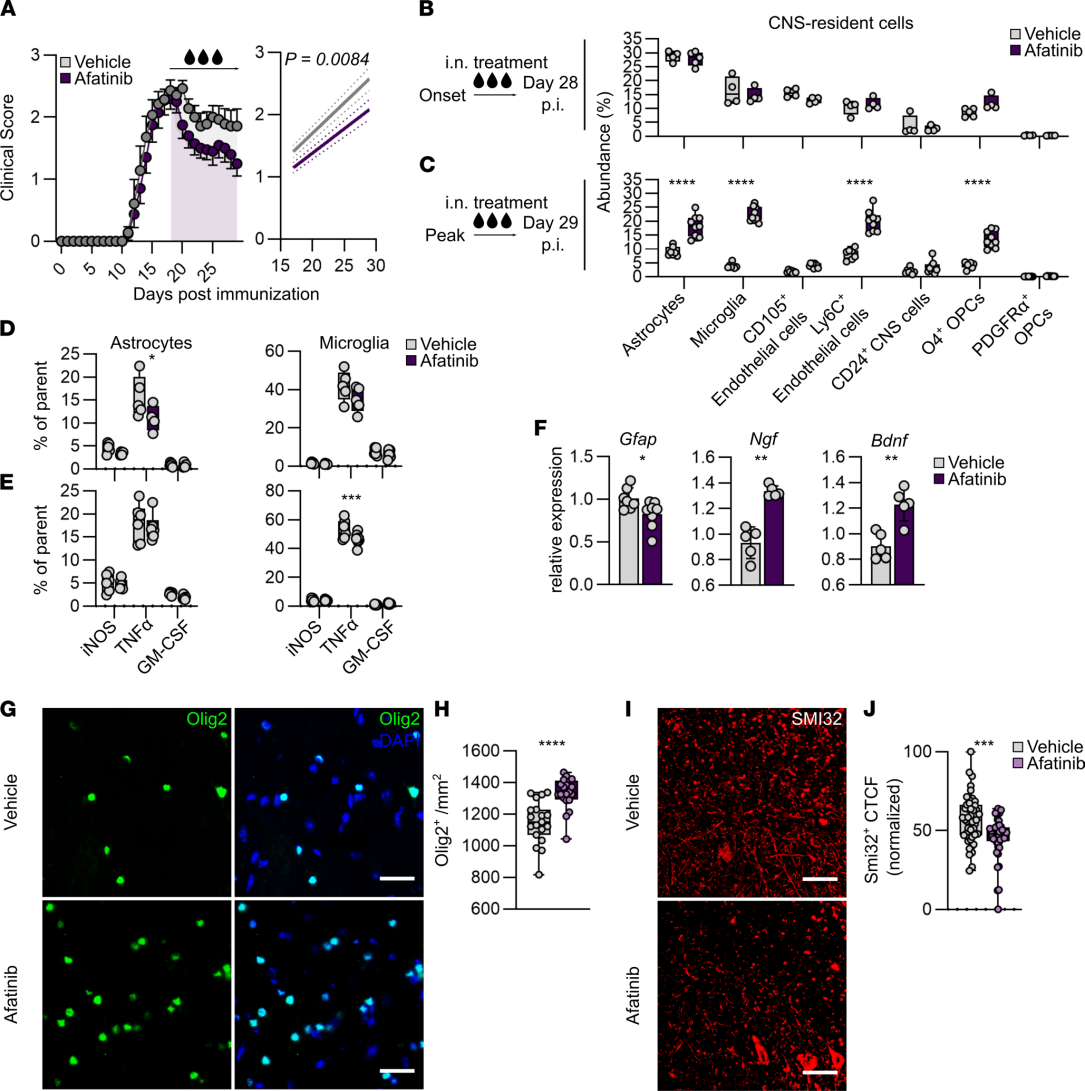
Therapeutic potential of i.n. afatinib application in chronic stages of CNS inflammation. (**A**) Clinical course (left) and linear regression analysis (right) of EAE in mice after daily i.n. treatment with vehicle or afatinib starting from peak of disease. The experiment was repeated twice. Vehicle (PBS), *n* = 7; afatinib, *n* = 12. Data are reported as mean ± SEM. (**B** and **C**) Abundance (percentage of total cell counts) of CNS cell populations during late-stage CNS inflammation in mice treated with vehicle or afatinib from symptom onset (vehicle, *n* = 4 or 5; afatinib, *n* = 4 or 5) (**B**) or peak of disease (**C**) (vehicle, *n* = 7; afatinib, *n* = 9), quantified by high-dimensional flow cytometry. Two-way ANOVA with Šidák’s multiple comparisons test. (**D** and **E**) Intracellular flow cytometry quantification of iNOS, TNF-α, and GM-CSF in astrocytes and microglia during late-stage CNS inflammation in mice treated with afatinib (*n* = 5) or vehicle (*n* = 5) from symptom onset (**D**) or peak of disease (**E**) (vehicle, *n* = 7; afatinib *n* = 8). Two-way ANOVA with Šidák’s multiple comparison test. (**F**) Relative expression of *Gfap, Ngf*, and *Bdnf* in ACSA2^+^ astrocytes during late-stage CNS inflammation in mice treated with afatinib (*n* = 8) or vehicle (*n* = 7) from peak of disease. Unpaired *t* test with Welch’s correction; data are reported as mean ± SD. (**G** and **H**) Representative fluorescence images (**G**) and quantification (**H**) of immunohistochemically labeled Olig2^+^ oligodendrocytes in lumbar spinal cord of mice treated with vehicle (*n* = 5) or afatinib (*n* = 5). Scale bars: 15 μm. Two-way ANOVA with Dunnett’s multiple comparisons test. (**I** and **J**) Representative fluorescence images (**I**) and corrected total cell fluorescence (CTCF) quantification (**J**) of axonal damage (SMI32^+^) in lumbar spinal cord of mice treated with vehicle (*n* = 5) or afatinib (*n* = 5). Two-way ANOVA with Dunnett’s multiple comparisons test. Scale bars: 15 μm. *P < 0.05; **P < 0.01; ***P < 0.001; ****P < 0.0001. p.i., postimmunization.

Both treatment strategies significantly ameliorated disease severity compared with vehicle-treated controls (Figure 5A, Figure 6A, and Supplemental Figure 8). Particularly, late-stage treatment increased the absolute number of CNS-resident cell populations (Figure 6, B and C), whereas CNS-infiltrating cells were only partially affected after afatinib treatment from peak of disease (Supplemental Figure 7C). This was concomitant with a reduced production of proinflammatory TNF-α by astrocytes after treatment with afatinib from symptom onset and a decrease in microglial TNF-α after treatment with afatinib from peak of disease (Figure 6, D and E). Albeit the absolute number of astrocytes was increased after i.n. delivery of afatinib from peak of disease, astrocytes were less activated and expressed increased levels of neuroprotective *Ngf* and *Bdnf* (Figure 6F). In addition, both treatment strategies effectively reduced the production of proinflammatory TNF-α, GM-CSF, or IFN-γ by CD45^hi^CD11b^+^ myeloid cells during late-stage inflammation, whereas the pathogenic activities of CD4^+^ effector T cells were particularly diminished after i.n. delivery of afatinib from symptom onset only (Supplemental Figure 7D).

Because CNS-infiltrating immune cells play a lesser role during late-stage CNS inflammation, when disease progression is primarily driven by CNS-intrinsic cells and their interactions, we next focused on the effects of afatinib on remyelination and axonal damage. Mice treated with afatinib had increased numbers of Olig2^+^ oligodendrocytes, which previously had been described to enhance remyelination in active and chronic MS lesions (17) (Figure 6G and Supplemental Figure 7, E and F). This was in line with reduced, ongoing axonal degeneration in afatinib-treated mice (Figure 6F and Supplemental Figure 7, E and G), together indicating that ErbB blockade by afatinib promotes regenerative mechanisms in late-stage CNS inflammation.

In conclusion, our data demonstrate that i.n. delivery of afatinib not only is effective in suppressing acute CNS inflammation but also suppresses late-stage chronic neuroinflammation, making it a potential candidate for the treatment of both acute and chronic progressive stages of autoimmune CNS inflammation in MS.

## Discussion

High-throughput transcriptomic and flow cytometry analyses have provided important insights into astrocyte heterogeneity in the context of neuroinflammation (7–9, 18–20). Although the increasing amount of data complements our understanding of the role reactive astrocytes play in MS pathogenesis, therapeutic strategies to therapeutically enhance protective astrocyte functions are limited. Here, we describe the identification of receptors and ligands associated with disease-stage-specific astrocyte activation states and our investigation of their therapeutic potential by pharmacological blockade using small molecules. We report that particularly afatinib, a CNS-penetrant small-molecule inhibitor, effectively suppressed mouse and human astrocyte proinflammatory responses and enhanced tissue-protective pathways to ameliorate acute and chronic autoimmune CNS inflammation by acting on CNS resident cells, including astrocytes, microglia, and oligodendrocytes.

We demonstrate that EGFR-dependent receptor–ligand interactions between astrocytes and CNS-infiltrating as well as CNS-resident cells are, among others, significantly regulated over the course of EAE. This finding is in line with those from previously published literature in which authors reported upregulation of ErbB signaling in astrocytes and other glial cells in multiple CNS pathologies (21), making ErbB signaling a relevant target for therapeutic intervention. Indeed, intravenous injection of an anti-human EGFR Ab demonstrated disease-ameliorating effects in a mouse model of RRMS (22). Although this finding strongly supports the notion that anti-EGFR therapy may be useful for the treatment of MS, our understanding of the immunological effects of EGFR blockade, particularly on glial cells, has remained limited in the context of CNS inflammation.

Here, we show that afatinib, a second-generation EGFR TKI effectively modulates pathogenic astrocyte functions in vitro and in vivo. Afatinib offers the distinct advantage over mAb therapy and other EGFR TKIs in that afatinib can be administered orally or i.n. and rapidly crosses the blood–brain barrier, where it can effectively modulate glial pathogenic activities. Administration of afatinib i.n. during early stages of CNS inflammation effectively ameliorated disease severity in acute stages of EAE, characterized by reduced infiltration of monocytes, the dominant cell type in active MS lesions and key drivers of acute CNS inflammation (1, 23, 24). In combination with transcriptional and functional in vitro data, these observations suggest that afatinib-mediated ErbB blockade in astrocytes down-modulates their capacity to attract inflammatory monocytes, consequently leading to reduced CNS inflammation and an improved disease outcome. In addition, i.n. application of afatinib during the late stage of EAE improved recovery, characterized by increased numbers of oligodendrocytes and reduced axonal damage. Both observations strongly correlate with disease progression and, collectively, suggest that i.n. administration of afatinib during late-stage CNS inflammation may promote remyelination (17, 25, 26). Albeit we cannot rule out a protective effect of afatinib on other cell types, our data consistently demonstrate a tissue-protective polarization of astrocytes in vitro and in vivo, while changes in the inflammatory capacity of CNS-resident and -infiltrating cell types such as microglia or T cells remain limited.

In summary, we have identified therapeutic strategies for the treatment of autoimmune inflammation in a mouse model of MS, which, at least to some extent, mediate their effect through the suppression of proinflammatory astrocyte responses and the induction of tissue-protective pathways relevant to acute and progressive disease stages. These findings indicate that particularly afatinib-mediated ErbB blockade may be an effective strategy for the therapeutic modulation of acute and chronic stages of MS.

## Methods

### Mice.

Mice (*n* = 2–5 animals per cage) were housed under a standard light cycle (12 hours of light, 12 hours of dark; lights on from 7 am to 7 pm) at 20°C to 23°C and humidity (~50%), with ad libitum access to water and food. Adult female mice 8 to 12 weeks old and P_0_–P_3_ pups were used on a C57Bl/6J background (The Jackson Laboratory, 000664).

### EAE.

EAE was induced in female C57Bl/6J mice 8 to 12 weeks old using 150 μg of MOG_35–55_ (Genemed Synthesis, 110582) mixed with freshly prepared CFA (using 20 mL of incomplete Freund’s adjuvant [BD Biosciences, BD263910] mixed with 100 mg of *Mycobacterium tuberculosis* H-37Ra [BD Biosciences, 231141] at a ratio of 1:1 [vol/vol at a concentration of 5 mg/mL]). All mice received 2 s.c. injections of 100 μL each of the MOG/CFA mix. All mice then received a single i.p. injection of pertussis toxin (List Biological Laboratories, 180) at a concentration of 2 ng/μL in 200 μL of PBS. Mice received a second pertussis toxin injection at the same concentration 2 days after EAE induction. Mice were monitored and scored daily thereafter. EAE clinical scores were defined as follows: 0, no signs; 1, fully limp tail; 2, hind limb weakness; 3, hind limb paralysis; 4, forelimb paralysis; and 5, moribund.

For treatment experiments, modulatory agents were administered i.n. starting after onset of first symptoms or at peak of EAE; 10 μL of the agents was applied drop by drop in each nostril. All agents were dissolved in PBS. The following concentrations were used: afatinib 10 mg/kg and pemigatinib 2.5 mg/kg. PBS was used as vehicle control.

### LPS-induced neuroinflammation.

Peripherally mediated neuroinflammation was induced as previously described (27, 28). In brief, 5 mg/kg LPS from *E*. *coli* O111:B4 (InvivoGen, tlrl-eblps) were dissolved in 200 μL of 1× PBS and injected i.p. into 8- to 12-week-old female C57Bl/6J mice. Sterile PBS served as the vehicle control. Mice were sacrificed after 24 hours, and ACSA2^+^ astrocytes were isolated from the cortex of LPS- and PBS-injected mice using the anti–ACSA-2 MicroBead Kit (Miltenyi, 130-097-678) after single-cell preparation, as described below.

### Intracerebroventricular injection of cytokines.

Acute cytokine-mediated neuroinflammation was induced in 8- to 12-week-old female C57Bl/6J mice by injection of 100 ng TNF-α (R&D, 410-MT) and 100 ng IL-1β (R&D, 401-ML) in 10 μL of PBS, or vehicle (PBS) into the lateral ventricle of each hemisphere, as previously described (8, 9). In brief, mice were anesthetized using 1% isoflurane mixed with oxygen. The head of each mouse was shaved and cleaned using 70% ethanol and lidocaine gel followed by a medial incision of the skin to expose the skull. The ventricles were targeted bilaterally using the coordinates ±1.0 (lateral), −0.44 (posterior), and −2.2 (ventral) relative to Bregma. Mice were injected i.c.v. with two 10-μL injections using a 10-μL Hamilton syringe (Sigma-Aldrich, 290 20787) on a Stereotaxic Alignment System (Kopf, 1900), sutured, and permitted to recover in a separate clean cage on a heating mat. Mice received an s.c. injection of 1 mg/kg meloxicam after i.c.v. injection and 48 hours later. Mice were sacrificed after 24 hours and ACSA2^+^ astrocytes were isolated from the cortex of cytokine- and vehicle-injected mice using the Anti-ACSA-2 MicroBead Kit (Miltenyi, 130-097-678) after single-cell preparation (as described below).

### CellPhoneDB analysis.

scRNA-Seq data were obtained from Wheeler et al. (8) and can be accessed under the SuperSeries accession numbers GSE130119, PRJNA544731, GSE118257, and GSE97942. CellPhoneDB receptor–ligand analysis was performed as described by Efremova et al. (11). In brief, statistical inference of ligand–receptor specificity was performed on count matrices by random permutation of cell clusters, followed by computation of the mean receptor–ligand expression value (R-L). The *P-*value cutoff was set to 0.001 for downstream analysis.

The absolute change of the R-L during the course of EAE was computed by subtracting (R-L)_naive_ from each stage (R-L)_stage_. Only receptor–ligand pairs that were present in all stages were considered. Visualization was performed using Seaborn and Matplotlib.

### Primary mouse astrocyte and microglia cultures and stimulation experiments.

Brains of mice aged P_0_ to P_3_ were dissected into PBS on ice. Brains of 6 to 8 mice were pooled, centrifuged at 500*g* for 10 minutes at 4°C and resuspended in 0.25% trypsin-EDTA (Thermo Fisher Scientific, 25200-072) at 37°C for 10 minutes. DNase I (Thermo Fisher Scientific, 90083) was added at 1 mg/mL to the solution, and the brains were digested for 10 more minutes at 37°C. Trypsin was neutralized by adding DMEM and GlutaMAX (Thermo Fisher Scientific, 61965026) supplemented with 10% FBS (Thermo Fisher Scientific, 10438026) and 1% penicillin/streptomycin (Thermo Fisher Scientific, 10500064), and cells were passed through a 70-μm cell strainer. Cells were centrifuged at 500*g* for 10 minutes at 4°C, resuspended in DMEM and GlutaMAX with 10% FBS and 1% penicillin/streptomycin, and cultured in T-75 flasks (Sarstedt, 83.3911.002) precoated with 2 μg/mL poly-l-lysine (Provitro, 0413) at 37°C in a humidified incubator with 5% CO_2_ for 5 to 7 days until confluency was reached.

Mixed glial cells were shaken for 30 minutes at 180 rpm, supernatant was aspirated, medium was changed, and the cells were shaken for at another 2 hours at 220 rpm. To increase purity, both the adherent astrocyte fraction and the shake-off fraction underwent CD11b^+^ magnetic cell separation (Miltenyi, 130-049-601).

Astrocytes (CD11b^–^ adherent cells) were seeded in culture plates, precoated with 2 μg/mL poly-l-lysine and prestimulated with 20 ng/mL TNF-α (R&D, 410-MT) and 20 ng/mL IL-1 (R&D, 401-ML) for 4 hours at 37°C. Microglia (CD11b^+^ cells) were seeded in culture plates, precoated with 2 μg/mL poly-l-lysine, and stimulated with 10 ng/mL LPS (from *E*. *coli* O111:B4; InvivoGen, tlrl-eblps) for 4 hours at 37°C. After 4 hours, modulators were added with or without the addition of fresh TNF-α (10 ng/mL), IL-1β (10 ng/mL), or LPS (10 ng/mL) at the following concentrations: afatinib (Tocris, 6812), 100 nM, 1 μM, 10 μM; dacomitinib (Sigma-Aldrich, PZ0330), 100 nM, 1 μM, 10μM; pemigatinib (Selleck Chemicals, S0088), 100 nM, 1 μM, 10 μM; erdafitinib (Selleck Chemicals, S8401), 100 nM, 1 μM, 10 μM; prosaptide TX14 (R&D, S8401), 100 nM, 1 μM, 10 μM; proglumide (Sigma-Aldrich, M006), 100 nM, 1 μM, 10 μM; ponatinib (Selleck Chemicals, S1490), 100 nM, 1 μM, 10 μM; GAS6 (R&D, 8310-GS-050), 3 ng/mL, 30 ng/mL, 300 ng/mL, as well as 30 ng/mL for microglia; PTN (R&D, 6580-PL-050), 1 ng/mL, 10 ng/mL, 100 ng/mL, as well as 50 ng/mL for microglia; MDK (R&D, 9760-MD-050), 1 ng/mL, 10 ng/mL, 100 ng/mL; MIF (R&D, 1978-MF-025/CF), 1 ng/mL, 10 ng/mL, 100 ng/mL, as well as 100 ng/mL for microglia; MIF-Iso1 (Sigma-Aldrich, 475837), 100 nM, 1 μM, 10 μM; and UNC2025 (Selleck Chemicals, S9662), 100 nM, 1 μM, 10 μM. For ACM experiments, microglia were seeded on in culture plates, precoated with 2 μg/mL poly-l-lysine and stimulated with ACM or medium control for 24 h.

### Primary human astrocyte cultures and stimulation experiments.

Primary human astrocytes were a gift of B. Winner, Department of Stem Cell Biology, University Hospital Erlangen, Erlangen, Germany, and originally were obtained from ScienCell (1800) and cultured according to the manufacturer’s instructions. In brief, cells were passaged in astrocyte medium (ScienCell, 1801) until confluency and subsequently plated onto plates precoated with 2 μg/mL poly-l-lysine (Provitro, 0413). For stimulation experiments, astrocytes were activated for 4 hours by stimulation with 20 ng/mL TNF-α (R&D, 210-TA-005) and 20 ng/mL IL-1β (R&D, 201-LB-005), 20 ng/mL IFN-γ (R&D, 485-MI-100/CF), 20 ng/mL IL-6 (R&D, 406-ML-005/CF), or 20 ng/mL GM-CSF (R&D, 415-ML-005/CF). After 4 hours, modulators were administered in addition to fresh TNF-α (10 ng/mL) and IL-1β (10 ng/mL), 20 ng/mL IFN-γ (R&D, 485-MI-100/CF), 20 ng/mL IL-6 (R&D, 406-ML-005/CF), or 20 ng/mL GM-CSF (R&D, 415-ML-005/CF) at the following concentrations: afatinib (Tocris, 6812), 10 μM; pemigatinib (Selleck Chemicals, S0088), 10 μM; and UNC2025 (Selleck Chemicals, S9662), 10 μM.

### RNA isolation.

After 24 hours of stimulation, primary astrocytes and microglia were lysed in 350 μL of RLT buffer (Qiagen) and RNA was isolated using the RNeasy Mini Kit (Qiagen, 74004) according to the manufacturer’s instructions. From each sample, 500 ng of RNA was transcribed into cDNA using the High-Capacity cDNA Reverse Transcription Kit (Life Technologies, 4368813). Gene expression was assessed by quantitative PCR (qPCR) using the TaqMan Fast Advanced Master Mix (Life Technologies, 4444556). The following TaqMan probes were used: *Gapdh* (Mm99999915_g1); *Actb* (Mm02619580_g1); *Gfap* (Mm01253033_m1); *Fgfr2* (Mm01269930_m1); *Gpr37l1* (Mm00661872_m1); *Mertk* (Mm00434920_m1); *Cd74* (Mm00658576_m1); *Erbb4* (Mm01256793_m1); *Nos2* (Mm00440502_m1); *Lif* (Mm00434762_g1); *Ccl2* (Mm00441242_m1); *Ccl3* (Mm00441259_g1); *Ccl5* (Mm01302427_m1); *Cxcl10* (Mm00445235_m1); *Tnf* (Mm00443258_m1); *Il6* (Mm00446190_m1); *Csf2* (Mm01290062_m1); *Ngf* (Mm00443039_m1); *Bdnf* (Mm04230607_s1); *NOS2* (Hs01075529_m1); *IL6* (Hs00174131_m1); *LIF* (Hs01055668_m1); and *CCL2* (Hs00234140_m1). qPCR data were analyzed by the ΔΔCt method.

For the collection of ACM, cells were washed extensively after 24 hours of stimulation and fresh medium was applied. The supernatant was collected after an additional 24 hours and centrifuged to remove cell debris.

### Intracellular flow cytometry of in vitro–stimulated primary astrocytes.

After 48 hours of stimulation, primary astrocytes were detached and washed once with cold 1× PBS. Live/dead staining was performed with LIVE/DEAD Fixable Aqua Dead Cell Stain Kit (Thermo Fisher Scientific, L34957) according to the manufacturer’s instructions. Surface staining was performed at 4°C in the dark for 20 minutes with Abs diluted in FACS buffer (1× PBS, 2% FBS, 2 mM EDTA). Cells were then washed twice with FACS buffer subsequently fixed for intracellular staining using the eBioscience Foxp3/Transcription Factor Staining Buffer Set (eBioscience, 00552300) according to the manufacturer’s instructions. The following Abs were used: BV421-CD11b (Biolegend, 101235), PE-Cy5.5-CD45 (Thermo Fisher Scientific, 35045180), PE-eFlour610-iNOS (eBioscience, 61592080), PerCP-eFlour710-TNF (eBioscience, 46732180), APC-GM-CSF (eBioscience, 17733182), and Ki67-AF700 (Biolegend, 652419).

Cells were acquired on a 3L Cytek Northern Lights flow cytometer. Analysis of flow cytometry data was performed with the OMIQ platform.

### Apoptosis assay of primary astrocytes and neuronal cells.

Primary mouse astrocytes were stimulated with afatinib (10 μM), UNC2025 (10 μM), or pemigatinib (10 μM) for 24 hours. N2A neuronal cells (American Type Culture Collection, CCL-131) were stimulated with ACM or control medium for 24 hours. Primary mouse astrocytes and N2A neuronal cells were detached and washed once in cold 1× PBS. Live/dead staining was performed with the LIVE/DEAD Fixable Aqua Dead Cell Stain Kit (Thermo Fisher Scientific, L34957) according to the manufacturer’s instructions. In addition, annexin V–propidium iodide staining was performed using the APC Annexin V Apoptosis Detection Kit with PI (Biolegend, 640932) according to the manufacturer’s instruction. Cells were washed once and resuspended in annexin V binding buffer before acquisition on a 3L Cytek Northern Lights flow cytometer. Analysis of flow cytometry data was performed with the OMIQ platform.

### Isolation of mouse splenic cells.

Spleens were mechanically dissected and dissociated by passing through a 100-μM cell strainer (Fisher Scientific, 10282631). Red blood cells were lysed with ACK lysing buffer (Life Technology, A10492-01) for 5 minutes and washed with 0.5% BSA and 2 mM EDTA at pH 8.0 in 1× PBS and prepared for downstream applications.

### Myeloid cell migration assay.

Splenic cells were isolated as described in the preceding paragraph and myeloid cells were purified by CD11b^+^ magnetic cell separation (Miltenyi, 130-049-601). Cells were seeded in the upper chamber of a 24-well cell-culture insert with a 5-μm pore size (Fisher Scientific, 10718502). The bottom chamber contained 600 μL of ACM. After 4 hours, nonmigrated cells in the upper chamber were removed carefully with a sterile swab. Migrated cells in the bottom chamber were detached and washed once in 1× PBS. Cells still in the process of migration were collected by incubation of the transwell insert in 100 μL TrypLE Select Enzyme (Thermo Fisher Scientific, 12563011) at 220 rpm for 10 minutes. After cells were washed, migrated and migrating cells were counted and acquired on a 3L Cytek Northern Lights flow cytometer.

### Isolation of cells from adult CNS.

Mice were perfused with cold 1× PBS and the CNS was isolated. Brain and spinal cord tissues were pooled in a 60-mm sterile culture dish and mechanically diced using sterile razors. Thereafter, diced tissue was transferred into 5 mL of enzyme digestion solution consisting of 37.5 μL of papain suspension (Worthington, LS003126) diluted in enzyme stock solution and equilibrated to 37°C. Enzyme stock solution consisted of 10 mL of 10× Earle’s Balanced Salt Solution (EBSS; Sigma-Aldrich, E7510), 2.4 mL of 30% d(+)-glucose (Sigma-Aldrich, G8769), 5.2 mL of 1 M NaHCO_3_ (VWR, AAJ62495-AP), 200 μL of 500 mM EDTA (Thermo Fisher Scientific, 15575020), and 168.2 mL of double-distilled water (ddH_2_O), filter sterilized through a 0.22-μm filter. Samples were shaken at 80 rpm for 40 minutes at 37°C. Enzymatic digestion was stopped with 5 mL of stop solution consisting of 1 mL of 10× high ovomucoid inhibitor solution and 20 μL of 0.4% DNase (Worthington, LS002007) diluted in 10 mL of inhibitor stock solution (ISS). The 10× high inhibitory ovomucoid ISS contained 300 mg of BSA (Sigma-Aldrich, A8806) and 300 mg of ovomucoid trypsin inhibitor (Worthington, LS003086) diluted in 10 mL of 1× PBS and filter sterilized using a 0.22-μm filter. ISS contained 50 mL of 10× EBSS (Sigma-Aldrich, E7510), 6 mL of 30% d(+)-glucose (Sigma-Aldrich, G8769), and 13 mL of 1 M NaHCO_3_ (VWR, AAJ62495-AP) diluted in 170.4 mL of ddH_2_O and filter sterilized through a 0.22-μm filter.

Tissue was mechanically dissociated using a 5-mL serological pipette and filtered through a 70-μm cell strainer (Fisher Scientific, 22363548) into a fresh, 50-mL conical tube. Tissue was centrifuged at 600*g* for 5 minutes and resuspended in 10 mL of 30% Percoll solution (9 mL of Percoll (GE Healthcare Biosciences, 17-5445-01), 3 mL of 10× PBS, 18 mL of ddH_2_O). Percoll suspension was centrifuged at 600*g* for 25 minutes with no breaks. Supernatant was discarded and the cell pellet was washed twice with 1× PBS, centrifuged at 500*g* for 5 minutes, and prepared for downstream applications. For analysis of astrocytes by reverse transcriptase qPCR, isolated CNS cells were subjected to ACSA2^+^ magnetic-bead separation using the Anti-ACSA-2 MicroBead Kit (Miltenyi, 130-097-678) according to the manufacturer’s instructions.

### Flow cytometry of CNS and splenic cells obtained from EAE animals.

Live/dead staining was performed with LIVE/DEAD Fixable Aqua Dead Cell Stain Kit (Thermo Fisher Scientific, L34957) according to the manufacturer’s instructions. Cells were subsequently stained at 4°C in the dark for 20 minutes with flow cytometry Abs, diluted in FACS buffer (1× PBS, 2% FBS, 2 mM EDTA). Cells were then washed twice with FACS buffer and resuspended in 1× PBS for acquisition. The following Abs were used in this study: BV421-CD11b (Biolegend, 101235); eF450-CD3 (Thermo Fisher Scientific, 48-0031-80); BV480-CD11c (BD, 565627); BV510-F4/80 (Biolegend, 123135); BV570-Ly6C (Biolegend, 128029); BV605-CD80 (BD, 563052); BV650-CD8 (Biolegend, 100741); PE-eFlour610-CD140a (Thermo Fisher Scientific, 61140180); SuperBright780-MHCII (Thermo Fisher Scientific, 78532080); BV711-CD74 (BD, 740748); PE-B220 (BD, 561878); PE-Ter119 (Thermo Fisher Scientific, 12592182); PE-Ly6G (Biolegend, 127607); PE-CD105 (Thermo Fisher Scientific, 12-1051-82); PE-Cy5-CD24 (Biolegend, 101811); PE-Cy7-CD31 (Biolegend, 102417); PerCP-eFlour710-CD86 (Thermo Fisher Scientific, 46086280); AF532-CD44 (Thermo Fisher Scientific, 58044182); PE-Cy5.5-CD45 (Thermo Fisher Scientific, 35045180); APC-Cy7-Ly6G (Biolegend, 127623); AF700-O4 (R&D, FAB1326N); BUV737-CD154 (BD, 741735); AF660-CD19 (Thermo Fisher Scientific, 606019380); and APC/Fire810-CD4 (Biolegend, 100479).

For intracellular flow cytometry staining, cells were fixed overnight after surface staining using the eBioscience Foxp3/Transcription Factor Staining Buffer Set (eBioscience, 00552300) according to the manufacturer’s instructions. For staining of intracellular cytokines, the following Abs were used: PE-eFlour610-iNOS (eBioscience, 61592080); PerCP-eFlour710-TNF (eBioscience, 46732180); APC-GM-CSF (eBioscience, 17733182); and AF700-Ki67 (Biolegend, 652419).

Cells were acquired on a 3L Cytek Northern Lights flow cytometer. Analysis of flow cytometry data was performed via the OMIQ platform. In particular, after import and scaling of fcs files, samples were down sampled to a normalized cell number. For dimensionality reduction, the opt-SNE tool was used (perplexity, 30; θ, 0.5, verbosity, 25), followed by Phenograph or UMAP clustering. Manual gating was performed according to Supplemental Figure 5C. Significance analysis of microarray (29) was performed on normalized MFI or abundance of cell clusters with 100 permutations and an FDR cutoff of 0.1.

### Mouse NGF and BDNF ELISA.

Primary mouse astrocytes were preactivated with TNF-α and IL-1β and stimulated as described above. After 24 hours, cells were extensively washed and fresh medium was applied. After another 24 hours, supernatant was collected and centrifuged to exclude cell debris. NGF and BDNF were measured in supernatants using the commercial mouse NGF (ScienCell, EK0470) and mouse BDNF (ScienCell, EK0309) ELISA kits according to the manufacturer’s instruction.

### Immunohistochemical staining and analysis.

For immunohistochemical analyses, mice were transcardially perfused with ice-cold 1× PBS. After perfusion, lumbar spinal cord (L1–L6) was dissected and processed for immunofluorescence labeling. The tissue was postfixed in 4% PFA/1× PBS for 24 hours. After postfixation, the spinal cords were dehydrated at 4°C in 30% sucrose in PBS overnight. Using liquid nitrogen–cooled 2-methylbutane, the tissue was frozen in tissue-Tek embedding medium and kept at –20°C for storage.

For the visualization and quantitative studies of Olig2-expressing oligodendrocytes and axonal damage (SMI32), 10-μm cross cryostat sections (Leica) of the spinal cords were obtained on glass slides and stored at –20°C. The spinal cords then subjected to immunohistochemistry for Olig2 and nonphosphorylated SMI32. The cross-sections were incubated in acetone for 10 minutes at –20°C for postfixation. After washing in 1× PBS for 5 minutes, the slides were incubated in blocking buffer (5% BSA, 10% donkey serum, 0.3% Triton-X, and 1× PBS) for 30 minutes. The following primary Abs were applied to the tissue and incubated overnight at 4°C: rabbit anti-Olig2 (1:200; Abcam, ab109186), mouse anti-SMI32 (1:1000; Biolegend, 801701) diluted in 1% BSA, 1% donkey serum, 0.3% Triton-X, and 1× PBS. On the following day, 3 washing steps of 5 minutes each preceded the incubation with the secondary Abs for 1 hour: donkey anti–rabbit IgG AF488 (1:500; Life Technologies, A21206) and donkey anti–mouse IgG AF647 (1:500; Dianova, 715-605-151). During the preincubation procedure, sections were washed 3 times for 5 minutes before and after 10 minutes of incubation with DAPI (1:100,000). After this process, coverslips were applied to the slides with Prolong Gold antifade and stored at 4°C for further analysis.

To evaluate immunohistochemical analyses, images of immunofluorescence-labeled sections were acquired using Zen 3.0 software (blue edition, Zeiss). For quantification, staining against Olig2 and SMI32 were examined in 4 distinct regions of the spinal cord (anterior column, posterior spinocerebellar tract, dorsal column, and gray matter) using a fluorescence microscope (Axio Observer Z1; Zeiss) at ×20 magnification. Olig2^+^ cells were quantified manually in an unbiased manner by the same investigator. The number of quantified cells was related to the area of spinal cord using the cell-counter plugin in ImageJ software (NIH). The areas of the evaluated spinal cord preparations were measured with ImageJ software. SMI32^+^ nonphosphorylated neurofilaments were measured using area-integrated intensity and mean gray value in the areas of interest. Data are shown as corrected total cell fluorescence calculated as follows: Integrated density – (area of interest × mean fluorescence of background readings). Image processing was performed using Photoshop CS6 (Adobe).

### Statistics.

Statistical examinations were carried out using GraphPad Prism 9. For analysis of multiple groups, 1-way ANOVA was applied. For multiple testing, a 2-way ANOVA with Dunnett’s or Šidák’s multiple comparisons test was applied. Family-wise significance and confidence level was set at *P* < 0.05. Additional information on the statistical tests used are provided in the figure legends.

### Study approval.

The animal studies were reviewed and approved by Bavarian State authorities (55.2.2-2532-2-1306, 55.2-2532.Vet.02-19-49).

## Author contributions

ML and VR designed the study, interpreted results, and wrote the manuscript. ML, LL, OV, TT, and AB performed experiments. TB and LN assisted in experiments. FP and UN helped with immunohistochemical staining. LL, OV, TT, AB, JBK, FJQ, and JW interpreted data and contributed to the manuscript.

## Supplementary Material

Supplemental data

## Version 1 04/08/2022

Electronic publication
