# Supplementary material for: Intranasal delivery of a small-molecule ErbB inhibitor promotes recovery from acute and late-stage CNS inflammation
Source: JCI Insight. 2022 Apr 8;7(7):e154824. doi: 10.1172/jci.insight.154824 (PMC9057609; doi:10.1172/jci.insight.154824)
Supplement: Supplemental data [file jciinsight-7-154824-s205.pdf]

**Supplementary Table 1.**

| Agent | Target | Indication |
|---|---|---|
| Afatinib | ErbB1, ErbB2, ErbB4 | Non-small cell lung cancer (30) |
| Dacomitinib | ErbB1, ErbB2, ErbB4 | Non-small cell lung cancer (31) |
| Erdafitinib | pan-FGFR, RET (c-RET), CSF-1R, PDGFR-α/PDGFR-β, FLT4, VEGFR-2 | urothelial cancer, metastatic or locally advanced bladder cancer (32) |
| Ponatinib | FGFR1, Abl, PDGFRα, VEGFR2 | Chronic myeloid leukemia, acute lymphoblastic leukemia (33) |
| Pemigatinib | FGFR1, FGFR2, FGFR3, FGFR4 | Advanced cholangiocarcinoma (34) |
| Proglumide | $CCK_{A,}$ $CCK_{B}$ | - |
| Prosaptide TX14(A) | GPR37L1 | - |
| UNC2025 | MERTK, FLT3 | - |
| MDK | ALK, LRP1, LRP2, PTPRZ1, NOTCH2 | - |
| PTN | ALK, PTPRZ1, LRP1, Nucleolin | - |
| MIF | CD74, CD44, CXCR2 | - |

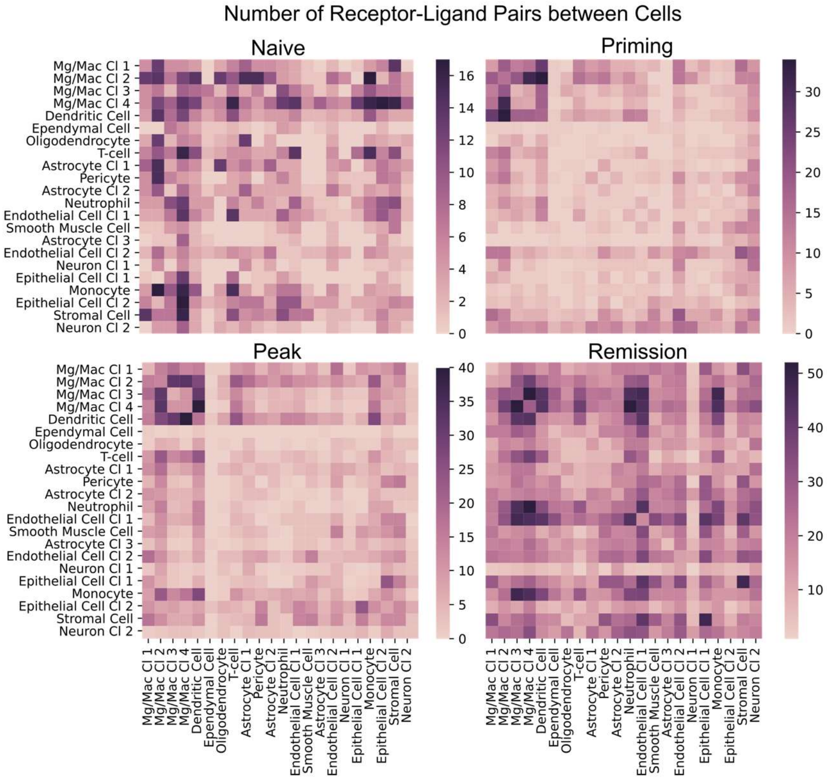


**Supplementary Figure 1. Aboslute number of cell-cell receptor-ligand interactions throughout EAE progression.** Number of inferred receptor-ligand pairs between cell types in the naïve, priming, peak and remission stage of EAE identified in scRNA-Seq by (8) after using the CellPhoneDB algorithm (11).

# Supplementary Figure 1

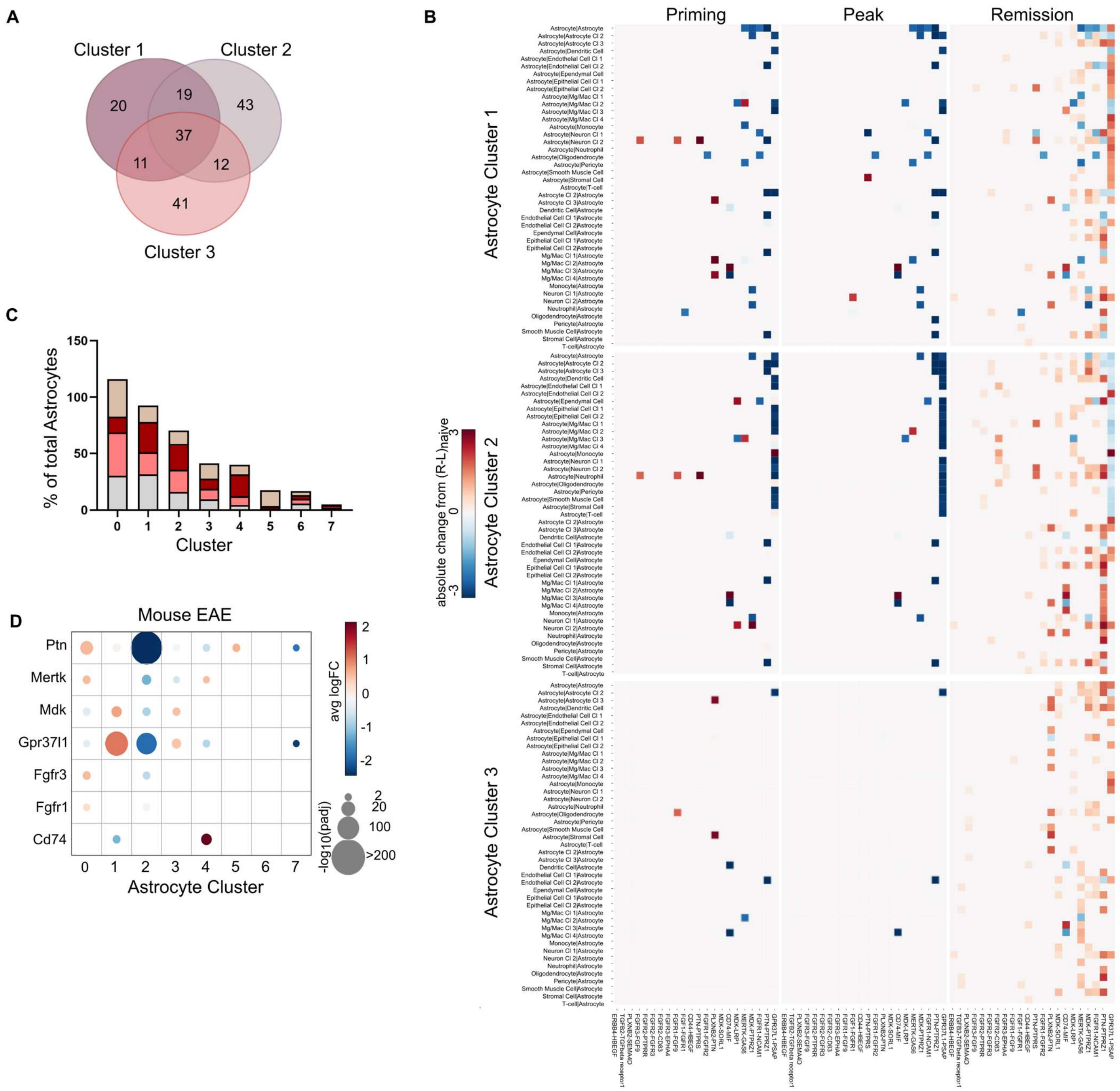


**Supplementary Figure 2. Inferred receptor-ligand interactions formed by astrocyte clusters during EAE.** (**A**) Venn diagram of receptor-ligand interactions formed by astrocyte clusters identified in Wheeler et al. (8). (**B**) Absolute changes ($(R\text{-}L)_{stage\ X}-(R\text{-}L)_{naïve}$) in co-expression of curated receptor-ligand pairs formed by astrocyte clusters throughout EAE. Positive changes indicate upregulation compared to naïve stage. (**C**) Abundance of astrocyte subclusters from (8) based on per cent composition in EAE and their expression of curated receptors and ligands (**D**).

# Supplementary Figure 2

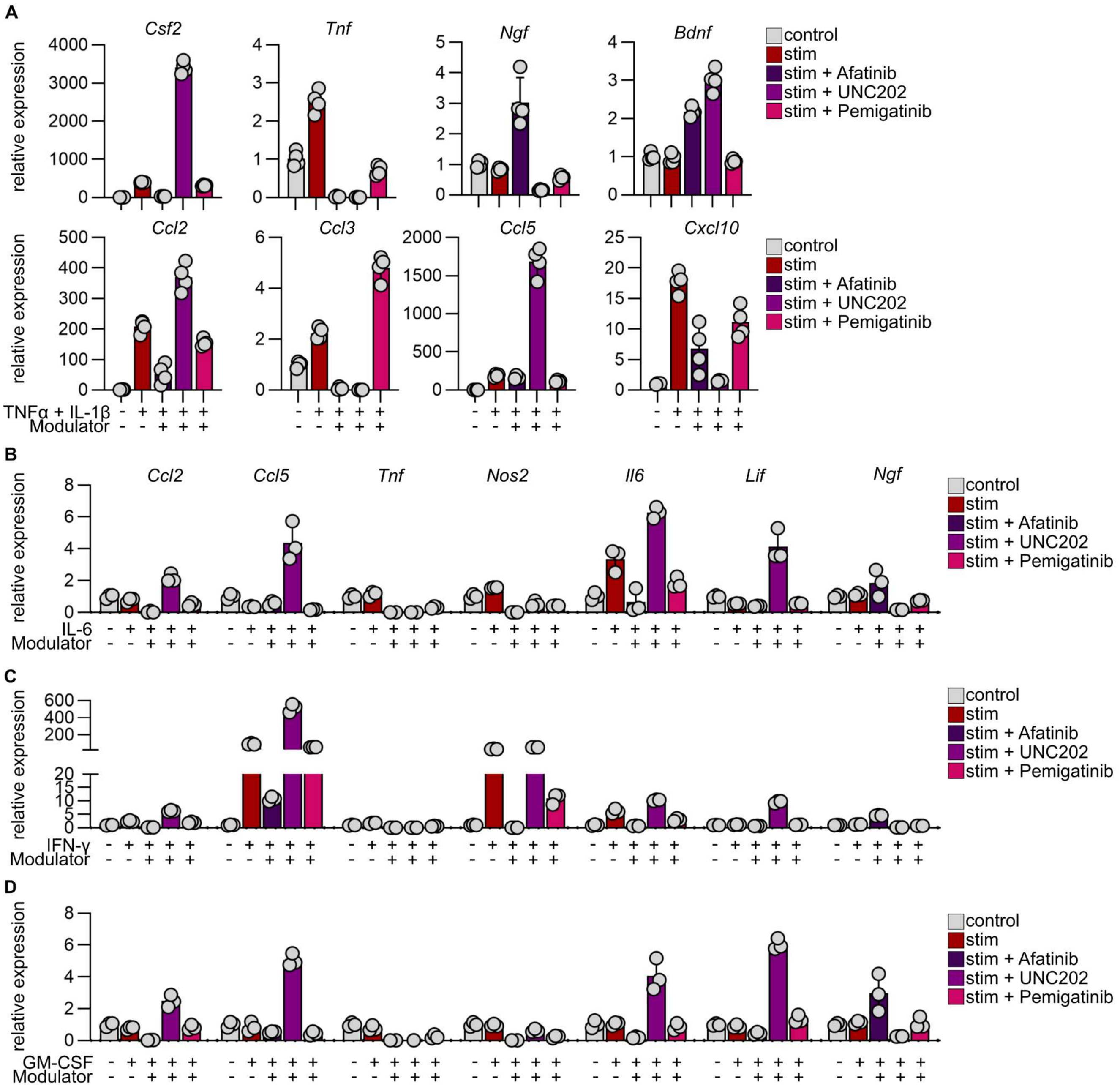


**Supplementary Figure 3. Afatinib, UNC2025, and Pemigatinib regulate inflammatory properties of astrocytes *in vitro*.** (**A**) Relative expression of *Csf2, Tnf, Ngf, Bdnf, Ccl2, Ccl3, Ccl5, Cxcl10* quantified by RT-qPCR in primary mouse astrocytes following stimulation with vehicle or TNFα + IL-1β ± Afatinib, UNC2025, Pemigatinib; n = 4 per group; Two-way ANOVA with Dunnett's multiple comparisons test. Data representative for n = 3 independent experiments. (**B**) Relative expression of *Ccl2, Ccl5, Tnf, Nos2, Il6, Lif,* and *Ngf* quantified by RT-qPCR in primary mouse astrocytes following stimulation with vehicle or IL-6, IFN-γ (**C**), or GM-CSF (**D**) ± Afatinib, UNC2025, Pemigatinib; n = 3 per group; Two-way ANOVA with Dunnett's multiple comparisons test.

# Supplementary Figure 3

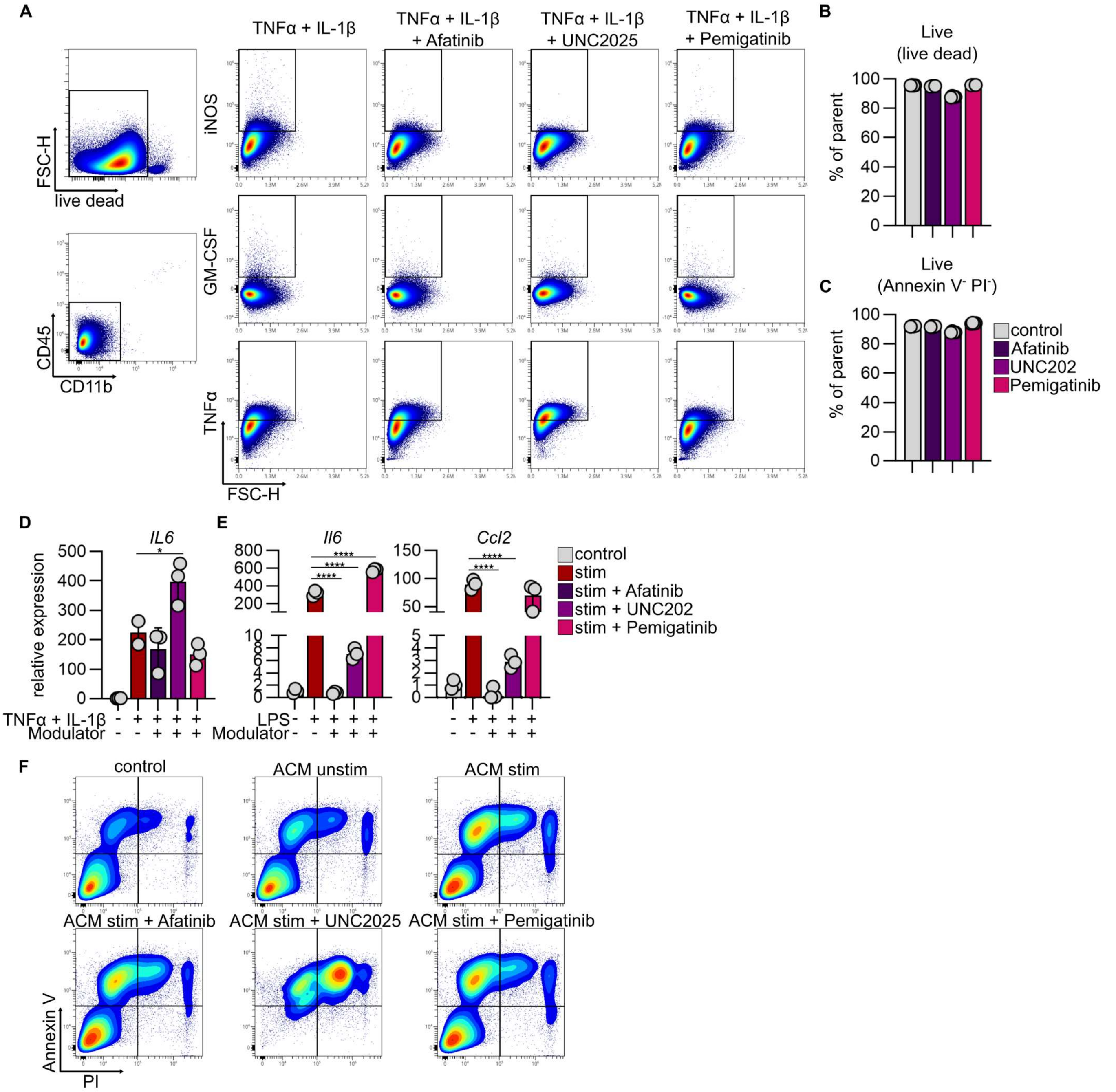


**Supplementary Figure 4. Functional assessment of Afatinib, UNC2025, and Pemigatinib on glial and neuronal cells *in vitro*.** (**A**) Representative scatter plots for intracellular flow cytometry measurements of TNFα, IL-1β, and GM-CSF in primary mouse astrocytes following stimulation with TNFα + IL-1β ± Afatinib, UNC2025 and Pemigatinib. (**B**) Quantification of amine-dependent and Annexin V (AV) / Propidium Iodid (PI) (**C**) live dead staining of primary mouse astrocytes following stimulation with vehicle, Afatinib, UNC2025 and Pemigatinib; n = 4 per group. (**D**) *IL6* expression quantified by RT-qPCR in human astrocytes ± stimulation with TNFα + IL-1β ± Afatinib, UNC2025 and Pemigatinib; n = 3 per group; One-way ANOVA with Dunnett's multiple comparisons test. (**E**) *Il6,* and *Ccl2* expression quantified by RT-qPCR in primary mouse microglia ± stimulation with LPS ± Afatinib, UNC2025 and Pemigatinib; n = 3 per group; One-way ANOVA with Dunnett's multiple comparisons test. (**F**) Representative scatter plots for Annexin V (AV) Propidium Iodid (PI) apoptosis assay of N2A neuronal cells following stimulation with astrocyte-conditioned medium (ACM). * $P < 0.05$; **** $P < 0.0001$.

# Supplementary Figure 4

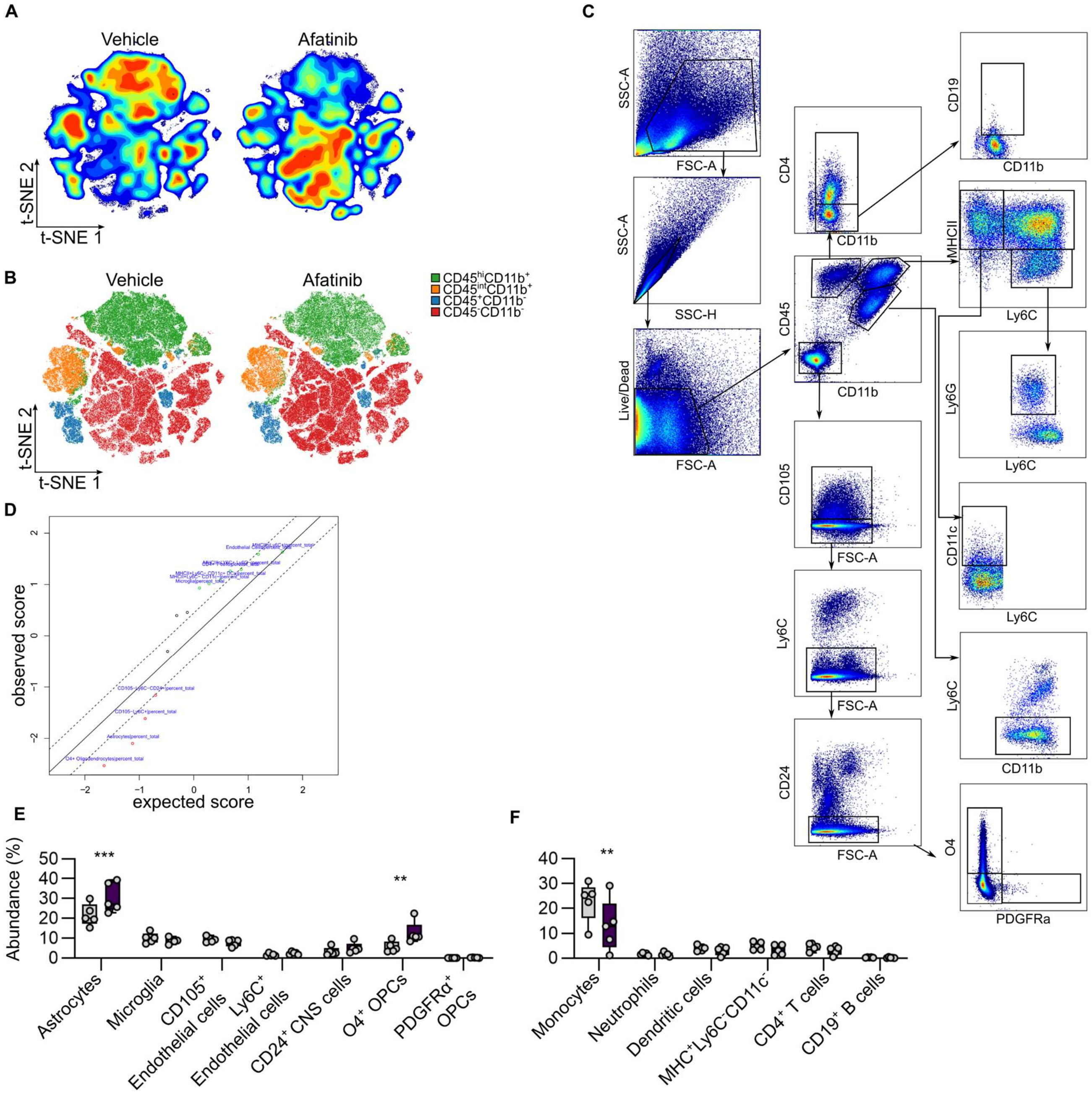


**Supplementary Figure 5. Intranasal delivery of Afatinib modulates cellular composition in the CNS during acute stages of EAE.** (**A**) Unsupervised clustering t-distributed stochastic neighbour embedding (t-SNE) plot of CNS cells during acute neuroinflammation obtained from mice treated intranasally with vehicle (n = 5) or Afatinib (n = 5) from symptom onset. (**B**) t-SNE plot with cellular clusters based on CD45 and CD11b expression of CNS cells obtained form mice treated with Afatinib (n = 5) or vehicle (n = 5) at peak of disease. (**C**) Gating strategy used to identify cell populations in the CNS based on their surface expression of cellular markers. (**D**) SAM plot of significantly differing cell populations in the CNS of mice treated with Afatinib (n = 5) or vehicle (n = 5) at peak of EAE. (**E**) CNS-intrinsic and -infiltrating (**F**) cell populations in the CNS of mice treated with Afatinib (n = 5) or vehicle (n = 5) at peak of EAE. Two-way ANOVA with Sidak's multiple comparisons test. ** $P < 0.01$; *** $P < 0.001$.

# Supplementary Figure 5

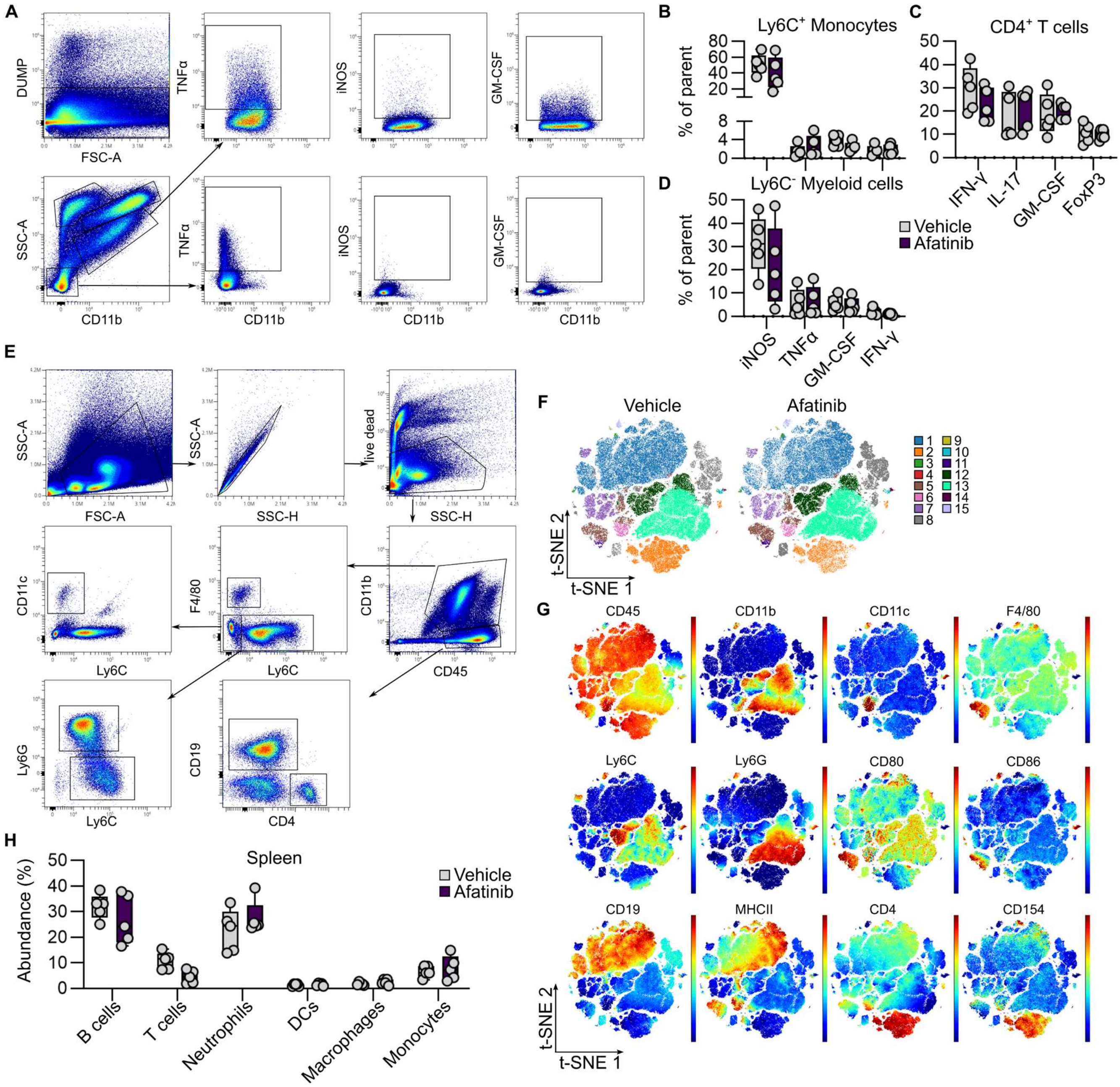


**Supplementary Figure 6. Intranasal delivery of Afatinib alters the inflammatory phenotype of cells in the CNS but not the periphery .** (**A**) Gating strategy used for the quantification of cytokines produced by microglia (top) and astrocytes (bottom) by intracellular flow cytometry. DUMP$^-$ cells (Ter-119$^-$, B220$^-$, CD140a$^-$, O4$^-$, Ly6G$^-$, Ly6C$^-$) cells were gating after exclusion of dead cells by amine-dependent live dead staining. (**B**) Relative expression of intracellular cytokines and intranuclear markers in CD45$^{hi}$CD11b$^+$Ly6C$^+$ monocytes, CD45$^+$CD11b$^-$CD4$^+$ T cells (**C**), and CD45$^{hi}$CD11b$^+$Ly6C$^-$ myeloid cells (**D**) in the CNS of mice treated with Afatinib (n = 5) or vehicle (n = 5) at peak of EAE measured by intracellular flow cytometry. (**E**) Gating strategy used to identify splenic cell populations based on their surface expression of cellular markers. (**F**) Unsupervised clustering t-distributed stochastic neighbour embedding (t-SNE) plot overlayed with clusters identified by FlowSOM-clustering of splenic cells measured by high-dimensional flow cytometry in mice treated intranasally with vehicle (n = 5) or Afatinib (n = 5) from symptom onset. (**G**) Median fluorescence intensity (MFI) of surface markers expressed by splenic cell clusters at peak of EAE. (**H**) Abundance of splenic cell populations in mice treated intranasally with vehicle (n = 5) or Afatinib (n = 5) from symptom onset following manual gating.

# Supplementary Figure 6

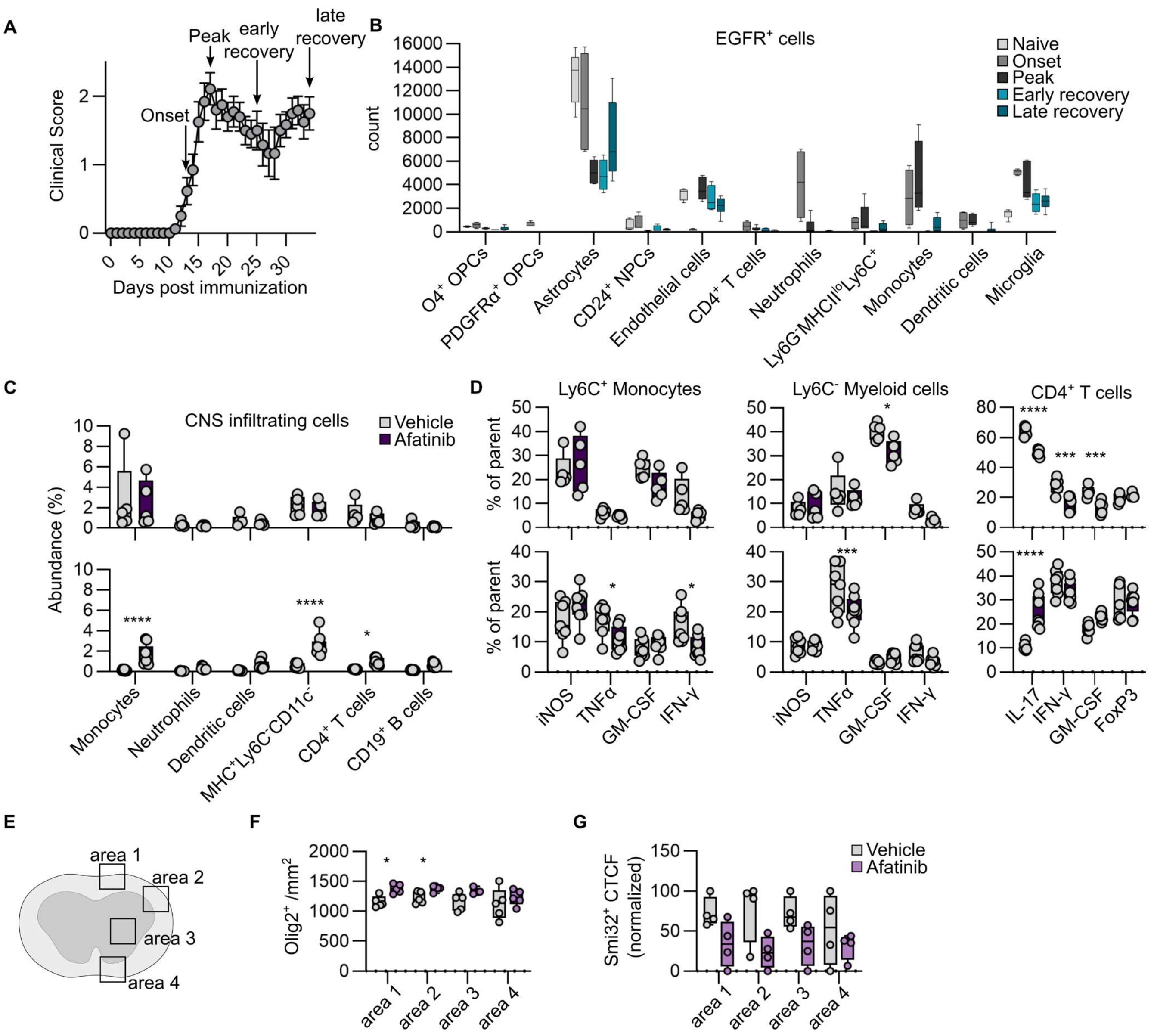


**Supplementary Figure 7. Intranasal delivery of Afatinib ameliorates late stage CNS inflammation .** (**A**) EAE development and clinical course in WT B6 mice; arrows indicate sampled timepoints. Data are shown as mean ± SEM. (**B**) Epidermal growth factor receptor (EGFR) expression on cell populations in the CNS of naïve mice (n = 5), during onset (n = 4), peak (n = 6), early- (n = 4) or late recovery (n = 8) stages of EAE, measured by high-dimensional flow cytometry. (**C**) Abundance of CNS infiltrating cells during late stage CNS inflammation in mice treated with vehicle or Afatinib from symptom onset (vehicle n = 5, Afatinib n = 5) (top) or peak of disease (vehicle n = 7, Afatinib n = 8) (bottom); Two-way ANOVA with Sidak's multiple comparisons test. (**D**) Relative expression of cytokines and intranuclear markers in $CD45^{hi}CD11b^{+}Ly6C^{+}$ monocytes, $CD45^{hi}CD11b^{+}Ly6C^{-}$ myeloid cells , and $CD45^{+}CD11b^{-}CD4^{+}$ T cells measured by intracellular flow cytometry in the CNS of  mice treated with vehicle or Afatinib from symptom onset (vehicle n = 5, Afatinib n = 5) (top) or peak of disease (vehicle n = 7, Afatinib n = 8) (bottom) during late stage CNS inflammation. Two-way ANOVA with Sidak's multiple comparisons test. (**E**) Schematic of the areas samples for IHC quantification in lumbar spinal cord sections. (**F**) Quantification of immunohistochemically labelled $Olig2^{+}$ oligodendrocytes and axonal damage ($SMI32^{+}$) in lumbar spinal cord of mice treated with vehicle (n = 5) or Afatinib (n = 5) during late stage CNS inflammation. Two-way ANOVA with Sidaks's multiple comparisons test. * $P < 0.05$; *** $P < 0.001$; **** $P < 0.0001$.

# Supplementary Figure 7

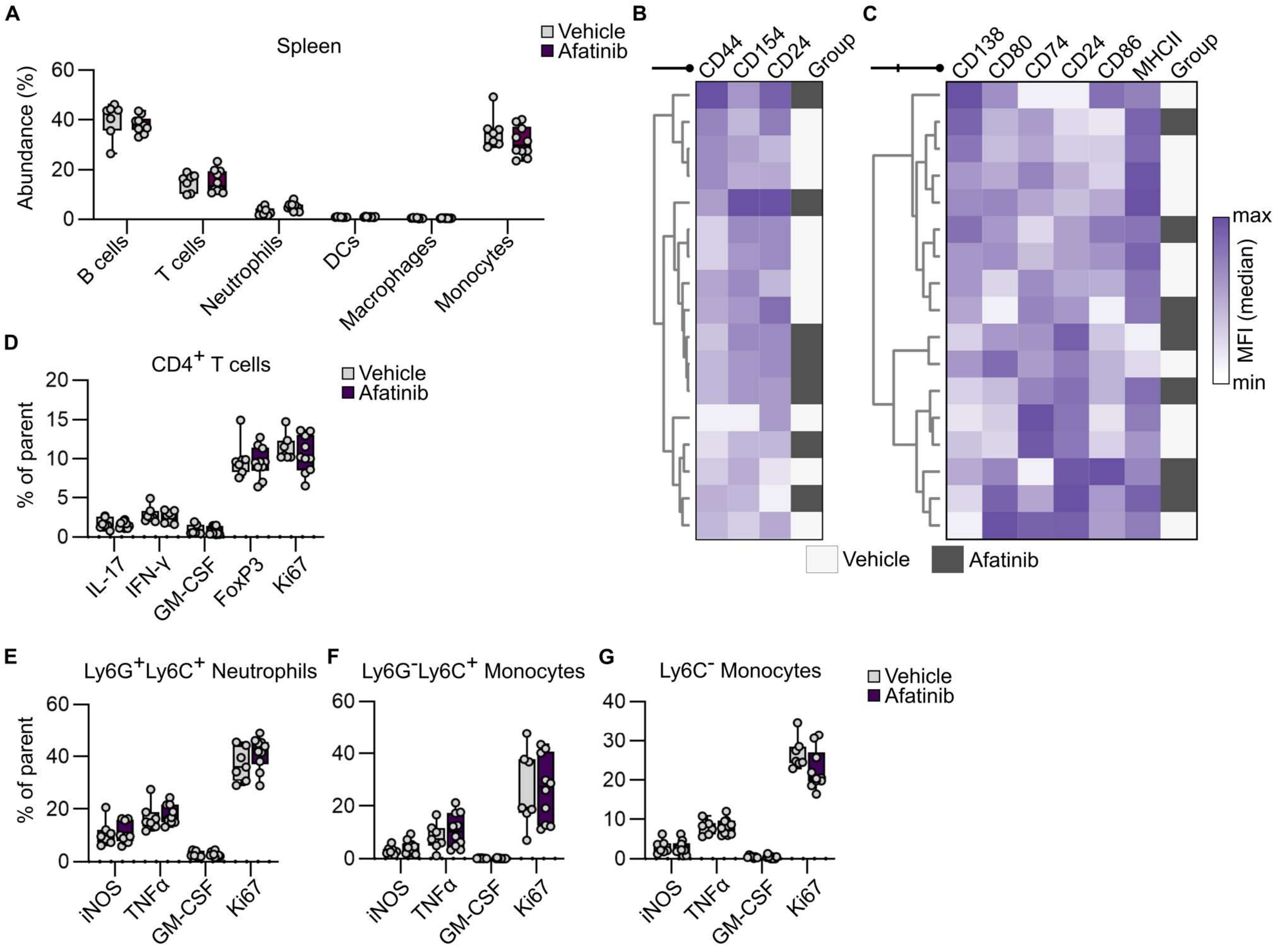


**Supplementary Figure 8. Effect of Afatinib on splenic cells during late stage CNS inflammation.** (**A**) Abundance of major immune cell populations in the spleen of mice treated with vehicle (n = 7) or Afatinib (n = 9) from peak of disease, measured by high-dimensional flow cytometry. (**B**) Eucledian clusterin based on normalized median fluorescence intensity (MFI) of activation markers expressed on T cells and myeloid cells (**C**) during late stage CNS inflammation in mice treated with vehicle (n = 7) or Afatinib (n = 9) from peak of disease, measured by high-dimensional flow cytometry. (**D**) Relative expression of cytokines and intranuclear markers during late stage CNS inflammation in $CD45^+CD11b^-$ $CD4^+$ T cells, $CD45^{hi}CD11b^+Ly6C^+Ly6G^+$ neutrophils (**E**), $CD45^{hi}CD11b^+Ly6C^+Ly6G^-$ monocytes (**F**), and $CD45^{hi}CD11b^+Ly6C^-$ myeloid cells (**G**) in spleens of mice treated with vehicle (n = 7) or Afatinib (n = 9) from peak of disease.

# Supplementary Figure 8
